# Spatial variation of rodenticides and emerging contaminants in blood of raptor nestlings from Germany

**DOI:** 10.1007/s11356-022-20089-1

**Published:** 2022-04-18

**Authors:** Alexander Badry, Detlef Schenke, Helmut Brücher, Nayden Chakarov, Thomas Grünkorn, Hubertus Illner, Oliver Krüger, Torsten Marczak, Gerard Müskens, Winfried Nachtigall, Ronald Zollinger, Gabriele Treu, Oliver Krone

**Affiliations:** 1 Department of Wildlife Diseases, Leibniz Institute for Zoo and Wildlife Research, Alfred-Kowalke-Straße 17, 10315 Berlin, Germany; 2 Institute for Ecological Chemistry, Plant Analysis and Stored Product Protection, Julius Kühn-Institut, Königin-Luise-Straße 19, 14195 Berlin, Germany; 3 Wiesenweihenschutz Brandenburg, Hauptstraße 11, 14913 Rohrbeck, Germany; 4 Department of Animal Behaviour, Bielefeld University, Morgenbreede 45, 33615 Bielefeld, Germany; 5 BioConsult SH, Schobüller Straße 36, 25813 Husum, Germany; 6 Arbeitsgemeinschaft Biologischer Umweltschutz/Biologische Station Soest, Teichstraße 19, 59505 Bad Sassendorf, Germany; 7 Independent, Bützow, Germany; 8 Müskens Fauna, van Nispenstraat 4, 6561 BG Groesbeek, The Netherlands; 9 Förderverein Vogelschutzwarte Neschwitz, Park 4, 02699 Neschwitz, Germany; 10 Natuurplaza, P.O. Box 1413, NL-6501 BK Nijmegen, The Netherlands; 11 Department Chemicals, Umweltbundesamt, Wörlitzer Platz 1, 06844 Dessau-Roßlau, Germany

**Keywords:** Biomonitoring, Birds of prey, Plant protection products, Rodenticides, Medicinal products

## Abstract

Wildlife exposures to pest controlling substances have resulted in population declines of many predatory species during the past decades. Many pesticides were subsequently classified as persistent, bioaccumulative, and toxic (PBT) and banned on national or global scales. However, despite their risks for non-target vertebrate wildlife, PBT substances such as anticoagulant rodenticides (ARs) are still permitted for use in Europe and have shown to threaten raptors. Whereas risks of ARs are known, much less information is available on emerging agrochemicals such as currently used PPPs and medicinal products (MPs) in higher trophic level species. We expect that currently used PPPs are relatively mobile (vs. lipophilic) as a consequence of the PBT criteria and thus more likely to be present in aqueous matrices. We therefore analyzed blood of 204 raptor nestlings of three terrestrial (red kite, common buzzard, Montagu’s harrier) and two aquatic species (white-tailed sea eagle, osprey) from Germany. In total, we detected ARs in 22.6% of the red kites and 8.6% of the buzzards, whereas no Montagu’s harriers or aquatic species were exposed prior to sampling. ΣAR concentration tended to be higher in North Rhine-Westphalia (vs. North-Eastern Germany) where population density is higher and intense livestock farming more frequent. Among the 90 targeted and currently used PPPs, we detected six substances from which bromoxynil (14.2%) was most frequent. Especially Montagu’s harrier (31%) and red kites (22.6%) were exposed and concentrations were higher in North Rhine-Westphalia as well. Among seven MPs, we detected ciprofloxacin (3.4%), which indicates that risk mitigation measures may be needed as resistance genes were already detected in wildlife from Germany. Taken together, our study demonstrates that raptors are exposed to various chemicals during an early life stage depending on their sampling location and underpins that red kites are at particular risk for multiple pesticide exposures in Germany.

**Supplementary Information:**

The online version contains supplementary material available at 10.1007/s11356-022-20089-1.

## Introduction

Agricultural intensification and associated chemical pollution resulted in environmental contamination and wildlife exposures over the past decades (Köhler and Triebskorn, 2013; Tang et al. 2021). Especially pest controlling substances have shown to persist in the environment, bioaccumulate in food webs, and reach toxic concentrations in predatory species (de Wit et al. 2020; Gómez-Ramírez et al. 2019; Kean et al. 2021). Raptors are particularly sensitive to anthropogenic pollution as many species have suffered from substantial population declines during the second half of the twentieth century (Helander et al. 2002; Shore and Taggart 2019). While numerous pesticides were consequently classified as persistent organic pollutants (POPs) and banned on a national or global scale during the 1970s and 1980s, residues of many POPs are still detectable in various species across Europe (de Wit et al. 2020; Kean et al. 2021). Under current European chemical legislations such as the Regulation on Biocidal Product Regulation (Regulation (EU) 528/2012) or Plant Protection Products (Regulation (EC) No. 1107/2009), substances are tested for persistent, bioaccumulative, and toxic (PBT) properties prior to their approval, which led to the elimination and restriction of already marketed substances. However, the identification of PBT properties is usually based on physicochemical properties and laboratory studies using aquatic, lower trophic level species such as fish (e.g., OECD No. 305). Studies on wildlife species, especially apex predators, are therefore important for adding information on chemical exposures of higher trophic level species under field conditions. Such information can then be used, e.g., in a weight of evidence approach for strengthening the connection between science and policy to ultimately improve chemical legislations (Wang et al. 2021).

Even though pesticides, i.e., biocides and plant protection products (PPPs), are assessed for PBT properties prior to their approval, certain known PBT compounds such as anticoagulant rodenticides (ARs) are still in use today due to a lack of suitable alternatives. The first generation of ARs was first introduced in the 1950s and subsequently supplemented by more persistent second-generation ARs (SGARs) due to the increasing resistance of rodents towards the first generation (Rattner et al. 2014). Today, ARs are registered in Germany as biocides to control populations of rodents in, e.g., urban areas and livestock farms, whereas their approval as PPPs (to protect agricultural crops) has expired and is only granted in exceptional cases. Due to their universal toxicity to vertebrate wildlife and potential to bioaccumulate in food webs, ARs are threatening raptors and other predators in Europe (Badry et al. 2021; Geduhn et al. 2015; Roos et al. 2021). In Germany, exposure to ARs has been shown to affect not only terrestrial compartments but also aquatic species, which was suggested to be related to their widespread use in sewer systems (Kotthoff et al. 2018; Regnery et al. 2019b, 2020).

Whereas exposure risks of many wildlife species to ARs and legacy PPPs are known, much less information is available on emerging agrochemicals such as currently registered PPPs and medicinal products (MPs) in higher trophic level species. Emission sources of currently used PPPs contrast those of legacy pesticides and comprise spray drift, agricultural surface runoff (Zhang et al. 2018), and direct exposures in the case of ground breeding birds (Bro et al. 2015). Recent studies analyzing liver residues indicated that raptors from Germany are exposed to currently used PPPs (Badry et al. 2021, 2022), whereas only limited information is available for PPPs other than neonicotinoids in raptor blood in Europe (Byholm et al. 2018; Rial-Berriel et al. 2020; Taliansky-Chamudis et al. 2017). For MPs, emission sources depend on their use as veterinary (VMP) or human medicinal product (HMP). Agriculturally related exposures to VMPs are for example linked to animal manure fertilization and scavenging on livestock, whereas HMPs enter the environment via wastewater or leaches from landfills (Shore et al. 2014; Wöhler et al. 2020). Both HMPs and VMPs were previously detected in liver and plasma of European raptors which included among others non-steroidal antiinflammatory drugs (NSAIDs) and antibiotics (Badry et al. 2021, 2022; Gómez-Ramírez et al. 2020).

All three contaminant groups (ARs, PPPs, and MPs) have been prioritized based on their respective risks for pan-European raptor monitoring (Badry et al. 2020). For investigating the extent of exposure of these three contaminant groups we focused on three terrestrial species, namely, the common buzzard *(Buteo buteo*, hereafter BUBT), the red kite (*Milvus milvus*, hereafter MIML), and the Montagu’s harrier (*Circus pygargus*, hereafter CIPY). BUBTs and MIMLs are both facultative scavengers that inhabit agriculturally influenced habitats such as forest patches and open grasslands (Heuck et al. 2013; Schindler et al. 2012), whereas CIPYs are ground nestling obligate hunters in, e.g., barley or wheat fields (Arroyo et al. 2002). The diet of all three species consists of small mammals depending on their abundance with a varying contribution of avian prey and invertebrates (reviewed in Badry et al. 2020). Besides terrestrial species, we also included both (semi-) aquatic raptors occurring in Europe, namely, the white-tailed sea eagle (*Haliaeetus albicilla*, hereafter HAAL) and the osprey (*Pandion haliaetus*, hereafter PAHA) as ARs, PPPs, and MPs were previously detected in aquatic species from Germany (Badry et al. 2022; Boulard et al. 2020; Kotthoff et al. 2018). Whereas PAHAs are exclusively foraging on fish, HAALs are mixed food web feeders that forage mainly on fish and waterfowl with a varying contribution of terrestrial carrion depending on season and availability (Nadjafzadeh et al. 2016).

The current work builds upon previous research investigating the exposure levels to ARs and agriculturally related substances in livers of avian apex predators from Germany (Badry et al. 2021, 2022). The analysis of apparently healthy nestlings was expected to overcome a potential sampling bias when analyzing internal organs of deceased individuals. Information on chemical exposures under field conditions is crucial to develop risk management measures for already identified PBT substances (i.e., ARs) and for supporting hazard assessments in European chemicals legislations. Specifically, we aim to (i) investigate the occurrence of currently used PPPs in blood as these substances are expected to be relatively mobile (vs. lipophilic) as a consequence of the PBT criteria and might therefore be present in rather aqueous matrices (i.e., blood). Furthermore, we aim to (ii) investigate the spatial contamination among the study populations as the exposure and associated risk factors for pesticide exposure (e.g., livestock farming and urbanization Badry et al. 2021; Geduhn et al. 2015)) differ among the sampling regions.

## Methods

### Sampling

**Fig. 1:**
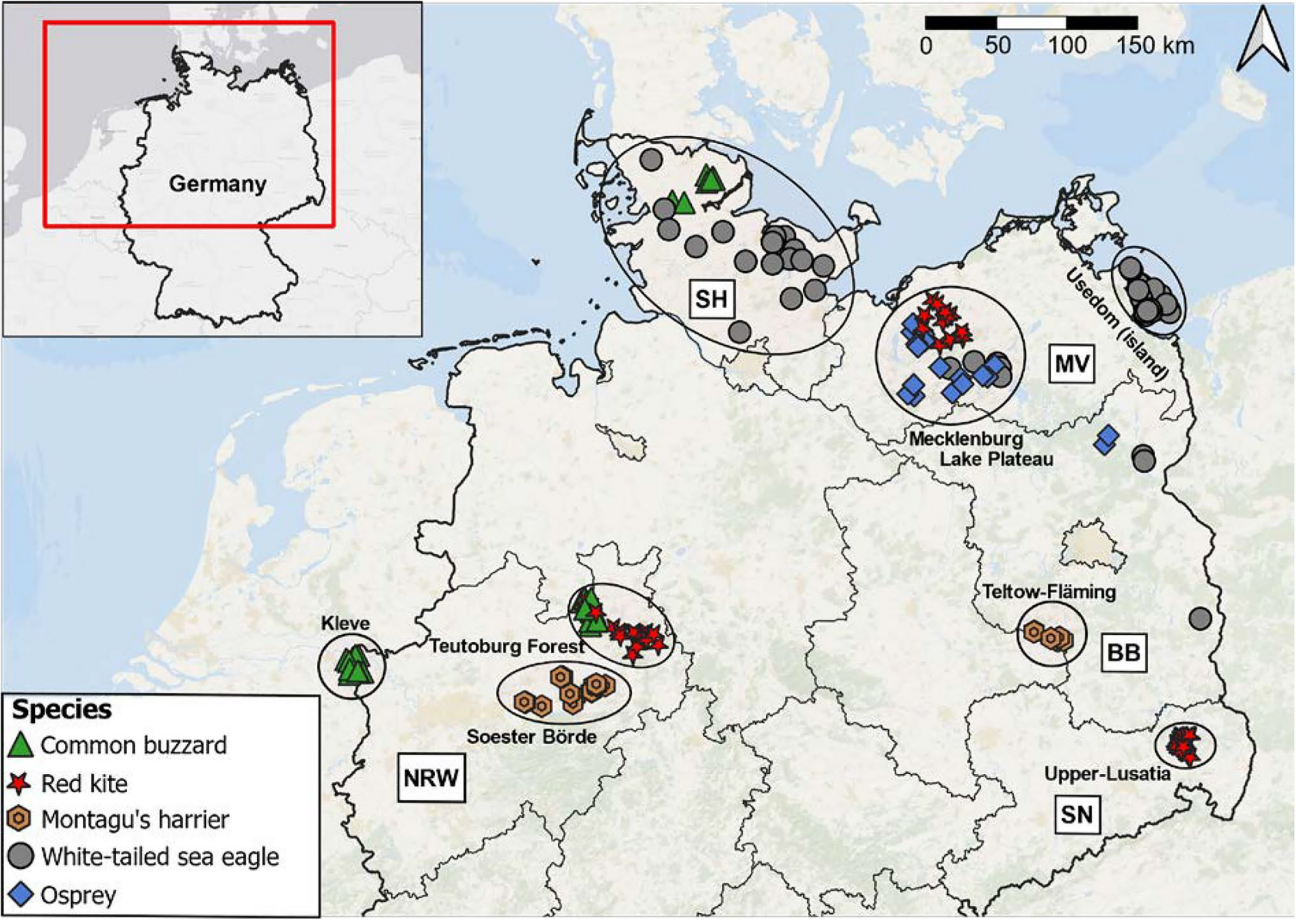
Sampling locations of the investigated raptor species within the federal states of Germany. Grey boxes refer to the abbreviations of the federal states: *NRW* North Rhine-Westphalia, *SH* Schleswig–Holstein, *MV* Mecklenburg-Western Pomerania, *BB* Brandenburg, *SN* Saxony. Green triangles refer to common buzzards (*Buteo buteo* (BUBT)), red stars to red kites (*Milvus milvus* (MIML)), brown doubled hexagons to Montagu’s harriers (*Circus pygargus* (CIPY)), grey circles to white-tailed sea eagles (*Haliaeetus albicilla* (HAAL)), and blue squares to ospreys (*Pandion haliaetus* (PAHA))

The sampling campaigns took place between May and August of 2019 and 2020 in Germany depending on the hatching dates and associated ringing dates of the five species (Fig. 1). The sampling of most nests was conducted when the nestlings were older than 3 weeks in order to reflect mainly dietary exposure routes (vs. potential maternal transfer). Biometric data (body weight, wing length) and reproductive status (number of nestlings per nest) are given in Table SI-1. One 0.7–1-mL blood sample per nest was taken from the *v. cutanea ulnaris* of one of the oldest/fittest nestlings during the local ringing campaigns to keep disturbances at the nest minimal. Blood sampling was conducted using sterile syringes with cannulas of 0.4–0.6-mm diameter. After sampling, we removed the cannula from the syringe and transferred the blood to K3EDTA Vacuette® containers. Most of the blood samples were frozen directly in the field whereas some samples were cooled using ice packs and frozen within 18 h after sampling (− 20 °C). When possible, we opportunistically searched for prey remains in the nests. In total, we took one blood sample from 204 nests from five raptor species in 2019 (*n* = 96) and 2020 (*n* = 108). The five species comprised the MIML (*n* = 53), BUBT (*n* = 35), CIPY (*n* = 29), HAAL (*n* = 64), and PAHA (*n* = 23). All CIPYs were sampled directly within cereal fields in approximately 50 × 50 m protection zones.

### Sampling locations

General information on land cover data classes of the sampling areas can be found in Figure SI-1. Briefly, the *BUBTs* were sampled mainly in three locations: the Teutoburg Forest area, the District of Kleve in North Rhine-Westphalia, and the northern parts of Schleswig–Holstein (Fig. 1). The Teutoburg Forest area is part of the central uplands in North Rhine-Westphalia (including the boarder region of lower saxony) and is influenced by mixed coniferous-deciduous forests, cereal fields in open areas, and livestock farming. The district of Kleve is located in North Rhine-Westphalia next to the border with the Netherlands and is influenced by livestock farming and agroforestry. Indications on the presence of intensive livestock farming are taken from the reported spatial sales of veterinary antibiotics in 2019 (Wallmann et al. 2020). The third sampling location, Schleswig–Holstein, is a federal state that is characterized by (field) agriculture, especially cereals and crops for fodder production as well as livestock farming. Furthermore, Schleswig–Holstein comprises various types of surface waters including rivers, lakes, and coastal waters of the North and Baltic Sea.

The *MIMLs* of the study were sampled in the Teutoburg Forest area in Western Germany, northern parts of Mecklenburg-Western Pomerania, and the Saxonian part of Upper Lusatia in Eastern Germany. Northern parts of Mecklenburg-Western Pomerania are characterized by similar agricultural types as Schleswig–Holstein with cereals being the dominant crop type followed by crops used for fodder production. The third sampling location in the Saxonian part of Upper Lusatia represents a rural area that is characterized by numerous small lakes of which some are used for aquaculture.

The *CIPYs* were sampled in the Soester Börde, a lowland region in the vicinity of the Teutoburger Forest area that is extensively used for growing cereals, mainly wheat, barley, maize, and rapeseed. A few samples were also taken from cereal fields in the Teltow-Fläming district in the south of Berlin (Eastern Germany), where field agriculture is also frequent.

The investigated *HAALs* originated from three sampling regions: the German part of the Baltic Sea island Usedom, the Mecklenburg Lake Plateau, and the federal state of Schleswig–Holstein. The island Usedom is characterized by mixed coniferous-deciduous and waterlogged forest as well as by Bodden and open coastal waters of the Baltic Sea. The Mecklenburg Lake Plateau, where also the *PAHAs* were sampled, has comparably low human population density and is characterized by a well-preserved landscape including a national park, numerous lakes, and mixed coniferous-deciduous forests.

### Selection of analytes

The selection of analytes followed the same rationale as in Badry et al. (2021) for liquid chromatography (LC)-mass spectrometry (MS)/MS compounds but included considerably more PPPs. In total, 90 PPPs (45 herbicides, 31 fungicides, 12 insecticides, 2 metabolites), of which 78 were approved during the start of the sampling campaign (05/2019), were included in the analysis (Table SI-2). Furthermore, the analysis included all currently registered ARs in Germany (brodifacoum, bromadiolone, chlorophacinone, coumatetralyl, difenacoum, difethialone, flocoumafen, and warfarin) as well as four widely used human medicinal products (ciprofloxacin, diclofenac, ibuprofen, sulfadiazine) and three veterinary antibiotics (enrofloxacin, marbofloxacin, sulfamethazine).

### Sample extraction and analysis 

The frozen blood samples were stored at − 80 °C after arrival at the analytical laboratory and were thawed before analysis. The sample treatment is presented step by step in Table SI-3. The blood samples (0.2 mL) were aliquoted in polypropylene tubes, spiked with a surrogate mixture for ongoing validation of analytical performance, and filled up to a final volume of 2 mL using acetonitrile. After adding a steel ball (Ø = 2 mm), we vortexed the samples and put them in an ultrasound bath for 5 min. After centrifugation (10 min, 5000 rpm), we transferred the aliquot to a new polypropylene tube. The procedure was repeated once by adding again 2 mL of acetonitrile and the supernatants were combined. Aliquots of 0.2 mL were then reduced to dryness and resuspended in internal standards and methanol/water for LC–MS/MS methods A, B, C, and E and in acetonitrile/water for method D (Table SI-4). After a brief ultrasound bath, the samples were filtrated through a syringe filter and stored at − 20 °C until analysis by LC–MS/MS.

The measurement of analytes was performed with a QTRAP-Triple Quad Linear Ion Trap 6500 + (SCIEX) in electrospray ionization mode. The identification and quantification of analytes were done with retention time and a precursor — product ion — transition (Table SI-5). For a multilevel calibration, we used 11 concentration levels from 0.01 to 20 pg µL^−1^. All analytes in all samples were quantified against a matrix-matched standard and the criterion for the acceptance of the calibration curve was the correlation coefficient (*r*^2^ > 0.99). The analyte concentrations were determined by the bracketing calibration method and calculated from the peak areas with the internal standards (Table SI-5). The calibration level with a relative standard deviation (RSD) below 20%, between the bracketing injections in a batch, was accepted as the lowest calibration level. The validation of the analytical procedure was checked by recovery tests using spiked pig blood (10, 100, and 1000 ng mL^−1^) stored in polypropylene tubes as well as in K3EDTA Vacuettes® (for rodenticides) to exclude potential effects of K3EDTA (used as anticoagulant in the blood sampling tubes) on rodenticide analysis. The mean recovery (*n* = 5) and the repeatability for each spike level are given in Table SI-6a. Additionally, we added surrogates to all samples (recovery and investigated samples) for ongoing validation of the analytical procedure. Mean recoveries and RSD of surrogate reproducibility are given in Table SI-6b. The pig blood samples, as well as the sample processing procedure, caused no detectable levels of the target analytes. The confirmation of the identity of an analyte was done with the linear ion trap mode with dynamic fill time. A substance was accepted when its enhanced product ion spectra in the sample (with intensity > 500 cps) matched more than 80% of those in the matrix standards in the same analysis sequence. All signals of confirmed analytes had a signal to noise ratio of > 6:1. The lowest calibration level of all batches was lower or equal to the calibration level to which the reporting limit (RL) refers. The measured concentrations of the analytes were neither surrogate nor recovery corrected.

### Spatial visualization and statistical analysis of contaminant data

All map-based visualizations were created using QuantumGIS software version 3.10.2 (QGIS Development Team 2020). We extracted all land cover classes in the sample area from the Corine Land Cover 2018 (EEA 2018) to visualize general land cover gradients (Figure SI-1). All other visualizations were created using the R package “ggplot2” (Wickham et al. 2016). We applied the non-parametric Mann–Whitney test (two-sided) using R version 4.1.2 (R Core Team. R 2021) for analyzing spatial differences in ΣAR and bromoxynil concentrations between terrestrial raptors (BUBT, MIML, CIPY) sampled in North Rhine-Westphalia, where intense cereal and livestock farming prevails, and terrestrial raptors sampled in North-Eastern parts of Germany (Geduhn et al. 2015; Wallmann et al. 2020) where population density is lower and intense agriculture less frequent (Figure SI-1). Concentration below the reporting limit was replaced with zero for statistical analysis and the level of significance was set to *p* < 0.05. No comparison among regions was possible for the (semi-) aquatic raptors (HAAL, PAHA) as both species are only resident in North-Eastern Germany. Concentrations are given as median (interquartile range: IQR) in ng mL^−1^ and refer to samples with detectable residues (Table SI-7), while “*n*” refers to the total sample number and “*n*^+^” to the number of nestlings that contained detectable contaminant residues in their blood.

## Results

**Fig. 2:**
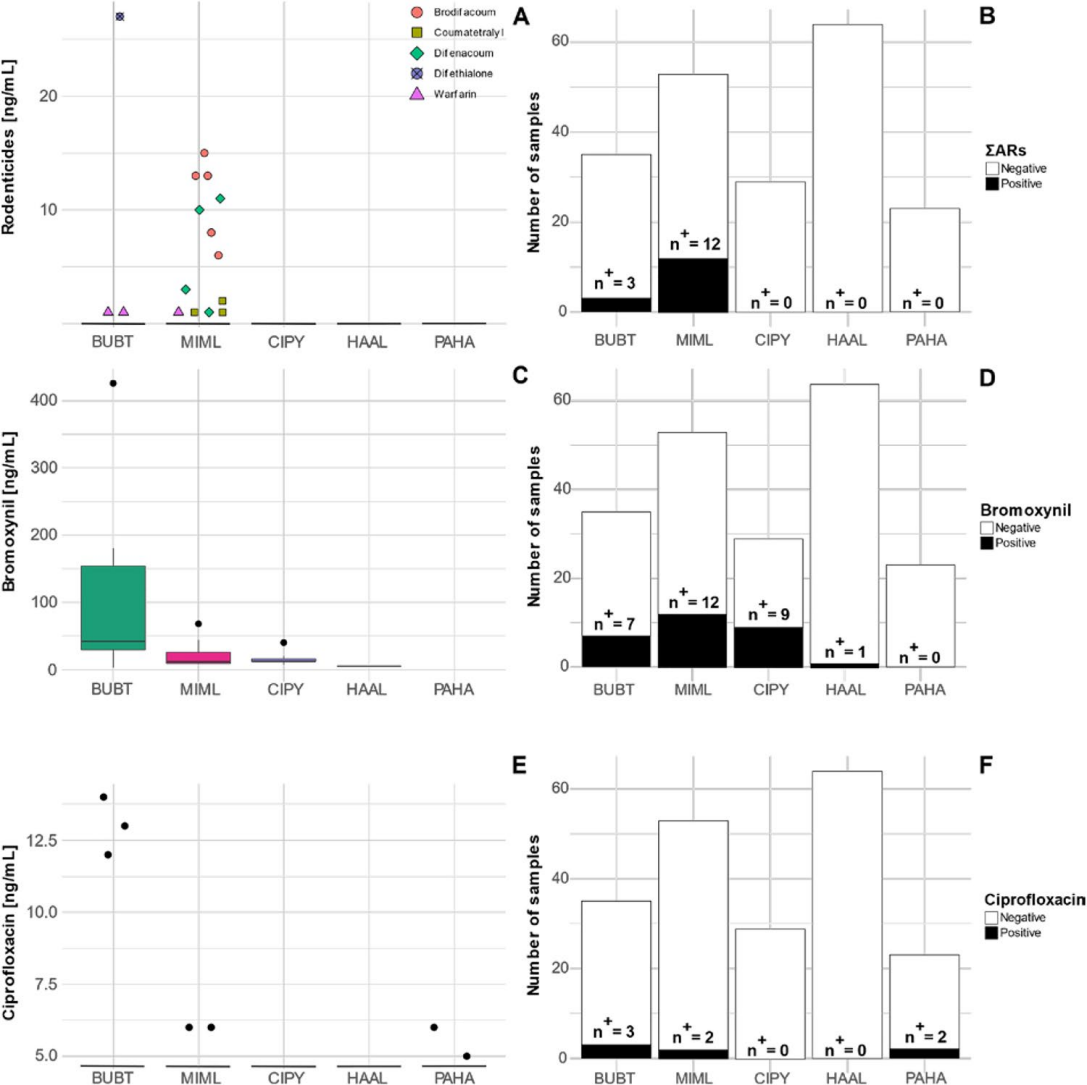
Concentrations of detected ARs (brodifacoum, difenacoum, difethialone, coumatetralyl, warfarin) (**A**) and ciprofloxacin (**E**) is given by dot plots and bromoxynil (**C**) given as boxplot (for samples > RL, reporting limit). The lower and upper hinges of the box correspond to the 25th and 75th percentile with the median given as horizontal line. The upper whisker extends from the hinge to the largest value no further than 1.5*IQR from the hinge. The lower whisker extends from the hinge to the smallest value at most 1.5*IQR of the hinge. The respective sample numbers (*n*) and samples with concentration > RL (*n*^+^) are given per species in **B**, **D**, and **F**. BUBT: *Buteo buteo* (common buzzard), MIML: *Milvus milvus* (red kite), CIPY: *Circus pygargus* (Montagu’s harrier), HAAL: *Haliaeetus albicilla* (white-tailed sea eagle), PAHA: *Pandion haliaetus* (osprey)

In total we detected five out of eight ARs (brodifacoum, difenacoum, difethialone, coumatetralyl, warfarin), six out of 90 PPPs (bromoxynil, fenpropidin, fenpropimorph, 2-methyl-4-chlorophenoxyacetic acid (MCPA), spiroxamine, terbuthylazine), and one out of seven MPs (ciprofloxacin) in our study (Fig. 2; Table SI-7).

### Anticoagulant rodenticides (ARs)

In total, we detected at least one AR in 7.4% (*n*^+^  = 15) of the 204 individuals (Fig. 2A, B). These ARs comprised brodifacoum (2.5%, *n*^+^  = 5, RL: 5 ng mL^−1^), difenacoum (2.0%, *n*^+^  = 4; RL: 2.5 ng mL^−1^), coumatetralyl (1.5%, *n*^+^  = 3; RL: 0.5 ng mL^−1^), warfarin (1.5%, *n*^+^  = 3, RL: 0.5 ng mL^−1^), and difethialone (0.5%, *n*^+^  = 1; RL: 2.5 ng mL^−1^), whereas bromadiolone (RL: 5 ng mL^−1^), chlorophacinone (RL: 10 ng mL^−1^), and flocoumafen (RL: 0.5 ng mL^−1^) were not detected in any of the blood samples.

The species with the highest detection rate of ΣARs was the MIML (22.6%, 7 (9.8) ng mL^−1^; Fig. 2A, B) with brodifacoum (9.4%, 13 (5) ng mL^−1^) being most frequently detected followed by difenacoum (7.6%, 6.5 (7.8) ng mL^−1^), coumatetralyl (5.7%, 1 (0.5) ng mL^−1^), and warfarin in one individual (1 ng mL^−1^). The only other species exposed to ARs was the BUBT (8.6%, 1 (13); Fig. 2B) which had residues of warfarin in two individuals (1 ng mL^−1^ each) as well as of difethialone (27 ng mL^−1^) in one individual. No AR residues were detected in CIPYs, HAALs, and PAHAs (Fig. 2B). The spatial visualization of ΣARs among the five species in 2019 and 2020 shows that in both years AR exposure occurred predominantly in MIMLs and BUBTs from North Rhine-Westphalia (Figure SI-2). This is furthermore supported by the Mann–Whitney test, where terrestrial raptors from North Rhine-Westphalia showed higher ΣAR contamination compared to terrestrial raptors sampled in North-Eastern Germany (*W* = 1879.5, *p*-value = 0.05).

### Plant protection products (PPPs)

**Fig. 3:**
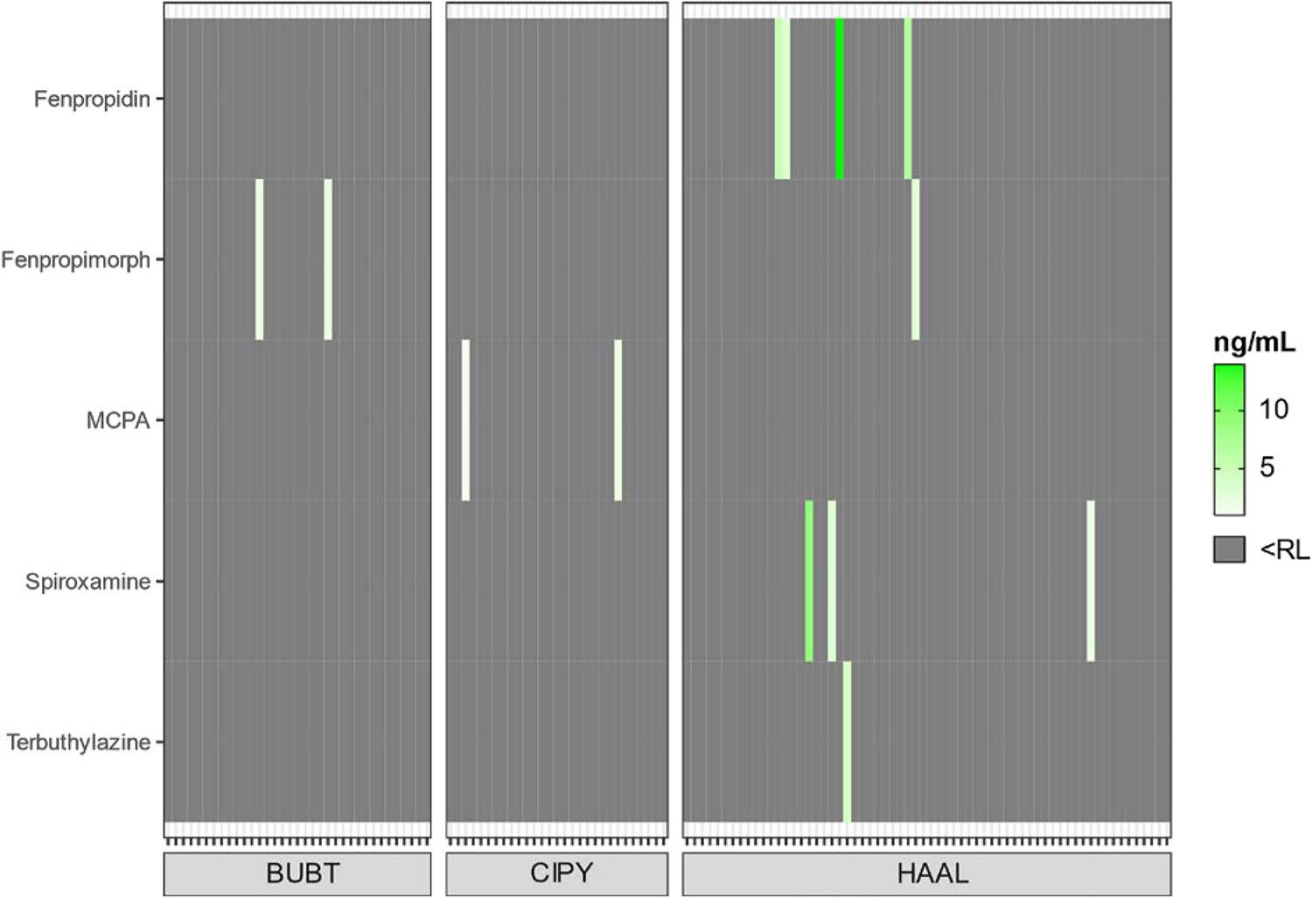
Heat map of detected PPPs other than bromoxynil (see Fig. 2) in blood of BUBT: *Buteo buteo* (common buzzard, *n* = 35), CIPY: *Circus pygargus* (Montagu’s harrier, *n* = 29), and HAAL: *Haliaeetus albicilla* (white-tailed sea eagle, *n* = 64) nestlings. Grey tiles in the heat map refer to samples below the reporting limit (RL) (Table SI-6a)

Among 90 analyzed PPPs, 82 were expected to be used in Germany at least in 2019 based on their sales figures (active substances; Table SI-2) and periods of grace of the four expired PPPs (epoxiconazole, fenpropimorph, pymetrozine, quinoxyfen). In total six PPPs were detected in the blood of the five investigated raptor species during 2019 and 2020 with bromoxynil showing the highest detection rate (14.2%, *n*^+^  = 29; Fig. 2D) followed by fenpropidin (2%, *n*^+^  = 4), fenpropimorph (1.5%, *n*^+^  = 3), spiroxamine (1.5%, *n*^+^  = 3), MCPA (1%, *n*^+^  = 2), and terbuthylazine (0.5%, *n*^+^  = 1) (Fig. 3). Among the five species bromoxynil exposure predominantly occurred in terrestrial raptors (MIML, BUBT, CIPY) from North Rhine-Westphalia (Figure SI-3). This is again supported by the Mann–Whitney test, where terrestrial raptors from North Rhine-Westphalia had significantly higher bromoxynil contamination compared to terrestrial raptors sampled in North-Eastern Germany (*W* = 1968, *p*-value < 0.05).

For bromoxynil, the highest detection rate occurred in CIPYs (31%, 12 (4) ng mL^−1^) followed by MIMLs (22.6%, 11.5 (16.8) ng mL^−1^), BUBTs (20%, 42 (124.5) ng mL^−1^), and one HAAL (5 ng mL^−1^) (Fig. 2C). No PPPs other than bromoxynil were detected in MIML and no PPPs at all were detected in blood of PAHAs. All residues of the fungicide fenpropidin were detected in HAALs (6.3%, 6 (4.3) ng mL^−1^), whereas fenpropimorph was detected in two BUBTs (2 ng mL^−1^ each) and in one HAAL (3 ng mL^−1^) (Fig. 3). Similar to fenpropidin, all spiroxamine residues were detected in HAALs (4.7%, 3 (3.5) ng mL^−1^), whereas MCPA was detected only in two CIPYs (1.5 (0.5) ng mL^−1^). Terbuthylazine was found only in one HAAL (4 ng mL^−1^) (Fig. 3).

### Medicinal products (MPs)

The only detected HMP was the fluoroquinolone antibiotic ciprofloxacin (RL: 5 ng mL^−1^) in 3.4% (*n*^+^  = 7) of the individuals, whereas diclofenac (RL = 1 ng mL^−1^), ibuprofen (RL = 5 ng mL^−1^), and sulfadiazine (RL = 0.5 ng mL^−1^) were not detected. Furthermore, none of the VMPs (enrofloxacin: RL = 2.5 ng mL^−1^, marbofloxacin: RL = 5 ng mL^−1^, sulfamethazine: RL = 0.5 ng mL^−1^) were detected.

Ciprofloxacin was detected in three BUBTs (8.6%; 13 (1) ng mL^−1^), two MIMLs (3.8%, 6 ng mL^−1^ each), and two PAHAs (8.7%, 6 and 5 ng mL^−1^) (Fig. 2 E and F). In contrast, no ciprofloxacin residues were detected in CIPYs and HAALs. All ciprofloxacin exposures occurred in 2019 in three BUBTs from Kleve in North Rhine-Westphalia as well as in two MIML and two PAHA from North-Eastern Germany (Figure SI-4).

## Discussion

### Anticoagulant rodenticides (ARs)

An increased risk for raptors and other predators to be exposed to ARs when preying on small mammals is well characterized and ARs have shown to be frequently detected in liver tissues of deceased raptors across Europe (López-Perea and Mateo, 2018) including Germany (Badry et al. 2021). ARs accumulate in livers where they exert their main mode of action by inactivating the vitamin K epoxide reductase (Rattner et al. 2014), whereas their half-lives in blood (< 2 days for chicken) are considerably lower (Horak et al. 2018). In Germany, most AR formulations consist of a single active ingredient and only a few formulations use combinations of two ingredients (e.g., difenacoum and brodifacoum) to overcome resistances in areas with high rodent infestation status (Regnery et al. 2019a). In the present study, we detected AR residues in blood of nestlings from only two terrestrial species, MIML and BUBT. A previous study reported AR residues in almost 15% of adult and nestling barn owls (*Tyto alba*) and common kestrels (*Falco tinnunculus*) from Spain, which was suggested to be related to a constant AR exposure through their prey (Rial-Berriel et al. 2020). The exposure rate of the terrestrial species from the current study was comparable (12.8%) but reporting limits for brodifacoum (5 ng mL^−1^), bromadiolone (5 ng mL^−1^), and chlorophacinone (10 ng mL^−1^) were higher compared to Rial-Berriel et al. (2020), which might have led to an underestimation of exposures for some ARs. Nevertheless, the investigated MIMLs in the present study had higher exposure rates (22.6%) and higher concentrations of the two most common ARs (brodifacoum and difenacoum) compared to the rodent predators from Spain in Rial-Berriel et al. (2020). This might be related to multiple exposure pathways of MIMLs as the species is a facultative scavenger and might have been exposed via foraging on sublethally exposed rodents as well as acutely poisoned rodents. Thus, our study emphasizes the particular risk of MIML for AR poisoning in Germany, which is in agreement with a study on liver samples from deceased MIMLs (Badry et al. 2021). Interestingly, the detection of ARs in blood of MIML nestlings predominantly occurred in the Teutoburger Wald (North Rhine-Westphalia, Western Germany), whereas the investigated MIMLs from Eastern (Saxony) and Northern Germany (Mecklenburg-Western Pomeranian) were exposed only once (Figure SI-2). The detection rate of BUBTs (8.6%) was lower compared to the MIML in our study. Interestingly the detection rate of BUBTs was similar to those in blood of juvenile red-tailed hawks (*Buteo jamaicensis*, *n* = 97), which represents the North American sister species of the European BUBT (Abernathy et al. 2018). Similar to the MIML, all exposures of BUBTs occurred in North Rhine-Westphalia as well, which may be attributed to higher anthropogenic influence (Figure SI-1) and intense livestock farming in the region (Wallmann et al. 2020). In general, ΣAR contamination in terrestrial raptors from North Rhine-Westphalia was higher compared to terrestrial raptors from North-Eastern Germany (*p* = 0.05). This is in agreement with a previous study on red foxes (*Vulpes vulpes*), where individuals were highly exposed to ARs in North Rhine-Westphalia as well (Geduhn et al. 2015). The diet of a rodent specialist, the barn owl, consisted around livestock farms in North Rhine-Westphalia mainly of non-target rodents from the taxon *Microtus* followed by *Sorex* spp. and *Apodemus* spp. (Geduhn et al. 2016). During the period (April–June) that coincides with our sampling campaign (May–July), rodents of the taxon *Apodemus* were the dominant prey items and regularly showed brodifacoum residues in their livers during baiting (Geduhn et al. 2016, 2014). Whether exposure pathways via foraging on non-target rodents such as *Apodemus* spp. represent a relevant exposure pathway for the investigated opportunistic raptors (MIML and BUBT) in our study area requires further investigation. However, foraging on rodents around livestock farms is considered to represent an important exposure pathway for both species based on their ecological traits (reviewed in Badry et al. 2020). In contrast to the MIML and BUBT, the current study did not detect AR residues in CIPYs, which was unexpected and might be related to foraging on non-rodent prey prior to sampling as CIPYs are opportunistic rodent predators depending on season and availability (Arroyo et al. 2002; Mirski et al. 2016). Furthermore, CIPY sampled in the current study nested directly in cereal fields, where the approval of ARs as PPPs (to protect agricultural crops) has expired and is only granted in exceptional cases. Interestingly, there was a population low of the common vole (*Microtus arvalis*) in the sampling area Soester Börde during 2019 and 2020 (HI, unpublished data), which might have resulted in an enhanced use of alternative prey such as birds and insects. However, as we are lacking systematic information on the diet prior to sampling we cannot disentangle whether their local foraging pattern or the ban of ARs as PPPs prevented CIPYs from exposures. However, this holds usually true for field studies in general, since dietary information, chemical exposure conditions, and information on the toxicokinetic behavior of a chemical (e.g., derived from laboratory study) are usually not assessable or unknown in field studies. An absence of ARs in blood of a raptor that is known to forage on small mammals was furthermore reported for eagle owls (*Bubo bubo*) from Spain, which was suggested to be related to the fast depletion of ARs in blood within days (Gómez-Ramírez et al. 2012). Therefore, new study designs using, e.g., consecutive blood samples from the same individual to cover a broader range of recent exposures in combination with the analysis of livers from deceased adult birds would help to evaluate the actual risk of ARs for CIPY and other raptors. In general, none of the investigated blood samples showed bromadiolone residues, which might contrast with results from other European countries (Italy, France, Netherlands, Romania) where bromadiolone was also registered as PPP until 31/05/2021 (Regnery et al. 2019a). Similar to CIPYs, no ARs were detected in nestlings of the investigated (semi-) aquatic species (HAAL and PAHA). Both species were sampled in North-Eastern Germany, where human population density is lower, and the intensification of agricultural land use is less pronounced compared to North Rhine-Westphalia (Figure SI-1). However, in Badry et al. (2021), 38% (*n* = 60) of the HAALs from North-Eastern Germany had AR residues in their liver but at lower concentrations compared to, e.g., the MIML. The absence of AR residues in the blood of HAAL nestlings and contradicting findings in livers of adults might be related to a combination of a generally lower AR contamination in North-Eastern Germany, shorter half-lives of ARs in blood, and the comparably high reporting limits for some of the targeted ARs in the present study. Furthermore, ARs accumulate over time with adults being at greater risk compared to juveniles (Badry et al. 2021; Roos et al. 2021), which might have further complicated their detection in nestlings. For the PAHA, the results of the current study are in line with Badry et al. (2021), where also no AR residues were detected in liver tissues of 13 PAHAs from a similar study region. These results indicate that piscivorous raptors in North-Eastern Germany might not be threatened by ARs, although the relatively small sample size (PAHA) limits an extrapolation on the population level. Further studies on aquatic predators (e.g., great cormorant (*Phalacrocorax carbo*), grey heron (*Ardea cinerea*), or Eurasian otter (*Lutra lutra*)) including prey species in highly populated areas as well as in areas of intensive livestock farming, such as North-Western Germany, might reveal further insights into potential biomagnification of ARs in aquatic food webs.

### Plant protection products (PPPs)

Recent studies targeting emerging contaminants (including PPPs) in raptor tissues such as liver and muscle detected a few PPPs as well as a few human and veterinary MPs, whereas the majority of target compounds were not detected (e.g., Badry et al. 2021; Sabater et al. 2020; Taylor et al. 2019). Similar results were obtained in the study on raptor blood by Rial-Berriel et al. (2020), where few currently approved and expired PPPs were detected. None of the detected PPPs in this study was detected by Rial-Berriel et al. (2020) where fenpropidin, fenpropimorph, spiroxamine, and terbuthylazine were also targeted. Whereas Rial-Berriel et al. (2020) detected the fungicide metrafenone (approved) in 2.7% of the blood samples, we did not detect metrafenone in our study. The most frequently detected PPP in our study was the herbicide bromoxynil, which was mainly found in the terrestrial raptors (BUBU, MIML, CIPY). Bromoxynil was approved during the study period in 2019 and 2020 but its approval expired (31/07/2021) due to a high risk for wild mammals from dietary exposures as well as for child residents (EC, 2020). In 2019, between 25 and 100 t of bromoxynil were sold in Germany (BVL 2020) for spraying it against broadleaved weeds (post-emergence) for miscanthus, alfalfa, red clover, grass (propagation), maize, and sorghum (EFSA 2018). Interestingly, bromoxynil contamination was significantly higher in terrestrial raptors (BUBT, MIML, CIPY) from North Rhine-Westphalia compared to those from North-Eastern Germany (Figure SI-3), which might be related to the intensive maize farming in, e.g., the Soester Börde and surrounding regions. Direct bromoxynil exposure to CIPY via spray application seems unlikely as concentrations were broadly similar to the tree nesting BUBT and MIML. A high risk for secondary poisoning was identified for bromoxynil octanoate, especially for earthworm-eating birds and mammals (EFSA 2017), which might explain exposures for BUBT and MIML as both species are known to forage on earthworms around the breeding time. Especially, the observed comparably high residues found in three BUBTs (127–426 ng mL^−1^) require further investigation with regard to bioaccumulation and potential adverse effects. For instance, although regulatory guidelines exist to ensure that commercial PPPs will not adversely affect bird populations, there are currently no test guidelines within the regulatory assessment specifically designed to evaluate bioaccumulation and biotransformation in birds (Kuo et al. 2022).

Other PPPs besides bromoxynil were detected at lower concentrations and detection rates. The currently approved fungicides fenpropidin (sold amount in 2019: 100–250 t) and spiroxamine (sold amount in 2019: 250–1000 t), as well as the approved herbicide terbuthylazine (sold amount in 2019: 250–1000 t; see BVL (2020)), were detected in only a few HAALs, whereas the other species were not exposed. Fenpropidin and spiroxamine are used via foliar spraying against fungal diseases of cereals (EFSA 2007, 2021), whereas terbuthylazine is applied via foliar spraying in maize and sorghum fields against annual and perennial grasses (EFSA 2019). These analytes were also included in the target screening of 30 HAAL livers using UHPLC-QTOF-MS/MS (Badry et al. 2022), where spiroxamine was detected in all individuals (LOD: 0.08 ng g^−1^). However, during the sampling period of the current study (spring-early summer), herbicides have shown to be more frequent, whereas fungicides are used later during the year to protect developed crops from fungal diseases (Brühl et al. 2021). Spiroxamine residues were furthermore detected in wild boar (*Sus scrofa*) and roe deer (*Capreolus capreolus*) muscles from Poland (Kaczyński et al. 2021), which are both common prey species of HAALs in Germany but not for the species analyzed in Rial-Berriel et al. (2020), where spiroxamine was not detected (LOQ: 0.1 ng mL^−1^). Terbuthylazine was one of the most frequently detected PPPs in insect traps from nature conservation areas in Germany (Brühl et al. 2021) and was previously detected in dermal swap samples from amphibians (Schenke et al. 2020) as well as in fecal samples (terbuthylazine‐2‐hydroxy) of Eurasian skylarks (*Alauda arvensis*) from Germany (Esther et al. 2022). Furthermore, terbuthylazine is formulated together with bromoxynil in one of the previously approved PPP products in Germany (Zeagran® ultimate), which indicates similarities in exposure pathways for both substances. Fenpropidin was detected in four and terbuthylazine in one HAAL, whereas no residues were detected in HAAL livers by Badry et al. (2022) (screening detection limit < 1.83 ng g^−1^) and blood of terrestrial raptors in Rial-Berriel et al. (2020) (LOQ: 0.1/0.4 ng mL^−1^), which might reflect matrix-specific differences in case of liver (vs. blood) as well as differences in feeding ecology compared to non-scavenging terrestrial raptors.

The only detected fungicide in a terrestrial raptor was fenpropimorph in two BUBTs. Similar to the other fungicides, fenpropimorph (sold amount in 2019: 100–250 t/a) was used via foliar spraying in, e.g., in cereal fields (EFSA, 2008), but its approval expired on 30/04/2019 (period of grace: 30/10/2020). Fenpropimorph residues have been previously reported in liver of a potential prey species (hedgehog: *Erinaceus europaeus*; Schanzer et al. 2021) of medium-sized terrestrial raptors from Germany. However, because no systematic or opportunistic dietary information was available for the nests of the exposed BUBTs, no conclusion can be drawn on potential sources and exposure pathways. Furthermore, we detected the herbicide MCPA (sold amount in 2019: 250–1000 t) in two CIPY nestlings sampled in cereal (barley) fields, where MCPA is applied from spring to early summer to control the growth of broadleaved weeds (EC, 2008). The absence of all targeted PPPs in PAHAs indicates that aquatic exposures via foraging on fish in inland habitats are probably not responsible for the observed exposures in HAALs. However, further systematic dietary investigations including exposure levels in prey species are needed to verify this assumption. Surprisingly, no PPPs other than bromoxynil were detected in MIMLs, which was unexpected as MIMLs are likely to be at risk for multiple exposures due to their opportunistic foraging behavior as facultative scavenger in agricultural landscapes. A limitation of the current study was that mainly parent compounds (i.e., dimethachlor) were analyzed although transformation products of PPPs (i.e., dimethachlor-oxa or ethiofencarb-sulfone) have shown to be present livers of HAALs (Badry et al. 2022). However, information on the metabolism of PPPs in avian wildlife including their distribution in internal organs and blood is scarce, which complicates the identification and selection of relevant metabolites.

### Human medicinal products (HMPs)

A previous target screening for 2441 contaminants in livers of deceased HAALs from Germany revealed that MPs (and transformation products) represented the majority of the detected compounds followed by legacy pollutants and PPPs (including transformation products) (Badry et al. 2022). Recently, wildlife species have been proposed as potential sentinels for detecting antimicrobial resistance in Germany due to their potential to act as reservoirs and dispersers of antimicrobial resistance genes (Plaza-Rodríguez et al. 2021). Among others, fluoroquinolones were prioritized within the critically important category for which risk management strategies are needed (WHO 2018). In the current study, we only detected the HMP ciprofloxacin in three BUBTs, two MIMLs, and two PAHAs in 2019 but not in 2020 (Figure SI-4). Sales of ciprofloxacin accounted for 32,980 t in 2009 (Bergmann et al. 2011) and ciprofloxacin is a known metabolite of enrofloxacin in mammals, where both show fast elimination in plasma of < 12 h after administration (Rao et al. 2002). Treated livestock or companion animals that were treated shortly before sampling might have therefore been a potential source of ciprofloxacin for the facultative scavengers (BUBT and MIML). Furthermore, ciprofloxacin was found in high levels in sewage sludge from Germany (Bergmann et al. 2011), which might have affected exposures of the terrestrial species as well. The comparably high concentrations in the BUBTs may be a cause of concern as experimental fluoroquinolone admission in bird eggs has shown to result in adverse effects on embryonic development (Hruba et al. 2019). Ciprofloxacin was furthermore reported in a HAAL liver from North-Eastern Germany (Badry et al. 2021), which was suggested to be related to aquatic exposures as ciprofloxacin is frequently detected in wastewater treatment plant effluents across Europe (Loos et al. 2013). However, no ciprofloxacin residues were found in blood of HAAL nestlings from this study but in blood from PAHA nestlings in North-Eastern Germany, which further indicates that aquatic exposure via fish might be the main update pathway. Taken together, our results demonstrate that terrestrial and aquatic exposure pathways for raptors to fluoroquinolones exist, which requires further investigation especially since the presence of ciprofloxacin resistance has already been reported for bacteria in wildlife from Germany (Plaza-Rodríguez et al. 2021). In contrast to fluoroquinolones, no residues of NSAIDs were detected in the current study, which contrasts observations in Badry et al. (2021), where ibuprofen residues were detected in 23.8% of HAAL livers as well two northern goshawks (*Accipiter gentilis*) and one MIML. However, half-lives of ibuprofen in blood are comparably short and peak after 1–2 h after administration in plasma of humans (Garrard 2014), which might explain why ibuprofen was not detected in the current study.

### Veterinary medicinal products (VMPs)

For veterinary antibiotics, sales are registered in Germany since 2011 and sales of the targeted veterinary fluoroquinolones (enrofloxacin and marbofloxacin) in 2019 were 4770 and 1155 t each (Wallmann et al. 2020). In contrast to our study, enrofloxacin was previously detected in a liver from a northern goshawk from Berlin as well as in a MIML that was either treated prior to death or foraged on treated prey items (Badry et al. 2021). Furthermore, enrofloxacin (but not ciprofloxacin (LOQ: 25 ng mL^−1^)) was detected in plasma of 29 griffon vulture nestlings (*Gyps fulvus*) from Spain, which was suggested to be related to foraging on livestock carcasses (Gómez-Ramírez et al. 2020). As previously discussed, the absence of enrofloxacin in blood of raptors in the current study may also be related to the short half-lives of fluoroquinolones in bird blood (Cox et al. 2004). In agreement with results for raptor livers in Badry et al. (2021), we did not detect the NSAID diclofenac. In contrast to Spain (see, e.g., Herrero-Villar et al. 2021), diclofenac is not used as VMP in Germany, which seems to protect facultative scavengers from exposure.

## Conclusion

Our study demonstrated that raptor nestlings are exposed to various ARs, PPPs, and one fluoroquinolone antibiotic across Germany, which is in agreement with previous studies on tissues of deceased raptors. However, a limitation of our study remains the final assessment of the analytical results, especially those below our reporting limits as toxicokinetic data, such as the half-life in blood of raptors, metabolization rates, and distribution behavior in raptors, are largely unknown.

Our results for ARs confirm previous observations in livers of deceased raptors demonstrating that MIMLs are at particular risk for AR exposure in Germany (Badry et al. 2021). Furthermore, BUBTs from the countryside have shown to be exposed to ARs as well, which indicates that urban BUBT populations might be at particular risk as shown for northern goshawks from Berlin (Badry et al. 2021). In general, AR exposure of both species seems to be more dominant in North Rhine-Westphalia, which might be related to the high population density and intense livestock farming in North-Western Germany. On the other hand, the absence of ARs in CIPY indicates that the ban of ARs as PPPs reduces exposures in cereal fields but further studies using consecutive blood samples and/or livers of adult birds are needed to confirm this observation. The absence of ARs in blood of HAAL and PAHA nestlings indicates that nestlings of piscivorous species living in lower populated areas such as North-Eastern Germany might not be at high risk for AR exposures.

Among the PPPs, bromoxynil was the most frequently detected substance and showed, similar to ARs, the highest concentration in terrestrial species from North Rhine-Westphalia. Further studies on acute and long-term effects on wildlife species should be investigated despite its withdrawal in 2021 since potential long-term risks from dietary exposure were identified for wild mammals in its final renewal report (EC, 2020). Other PPPs such as spiroxamine, fenpropidin, or fenpropimorph were only occasionally detected in a few individuals, whereas the majority of the targeted PPPs was not detected. However, some fungicides might have been applied during later stages of our sampling campaigns (Brühl et al. 2021), which calls for further investigations on fungicide exposures during summer since, e.g., spiroxamine has shown to be frequently detected in livers of deceased HAALs (Badry et al. 2022). For MPs, the detection of the fluoroquinolone ciprofloxacin in BUBTs, MIMLs, and PAHAs calls for general risk mitigation measures to reduce the environmental impact of antibiotics in the environment as resistance genes were already detected in wildlife from Germany (Plaza-Rodríguez et al. 2021).

## Supplementary Information

Below is the link to the electronic supplementary material.

Supplementary file1 (PDF 1477 KB)

## Data Availability

The datasets used and/or analyzed during the current study are available from the corresponding author on reasonable request.
