# Correction to: Spatial variation of rodenticides and emerging contaminants in blood of raptor nestlings from Germany

**DOI:** 10.1007/s11356-022-23702-5

**Published:** 2022-10-20

**Authors:** Alexander Badry, Detlef Schenke, Helmut Brücher, Nayden Chakarov, Thomas Grünkorn, Hubertus Illner, Oliver Krüger, Torsten Marczak, Gerard Müskens, Winfried Nachtigall, Ronald Zollinger, Gabriele Treu, Oliver Krone

**Affiliations:** 1 Department of Wildlife Diseases, Leibniz Institute for Zoo and Wildlife Research, Alfred‑Kowalke‑Straße 17, 10315 Berlin, Germany; 2 Institute for Ecological Chemistry, Plant Analysis and Stored Product Protection, Julius Kühn-Institut, Königin‑Luise‑Straße 19, 14195 Berlin, Germany; 3 Wiesenweihenschutz Brandenburg, Hauptstraße 11, 14913 Rohrbeck, Germany; 4 Department of Animal Behaviour, Bielefeld University, Morgenbreede 45, 33615 Bielefeld, Germany; 5 BioConsult SH, Schobüller Straße 36, 25813 Husum, Germany; 6 Arbeitsgemeinschaft Biologischer Umweltschutz/Biologische Station Soest, Teichstraße 19, 59505 Bad Sassendorf, Germany; 7 Independent, Bützow, Germany; 8 Müskens Fauna, van Nispenstraat 4, 6561 BG Groesbeek, The Netherlands; 9 Förderverein Vogelschutzwarte Neschwitz, Park 4, 02699 Neschwitz, Germany; 10 Natuurplaza, P.O. Box 1413, NL‑6501 BK Nijmegen, The Netherlands; 11 Department Chemicals, Umweltbundesamt, Wörlitzer Platz 1, 06844 Dessau‑Roßlau, Germany

**Correction to: Environmental Science and Pollution Research (2022) 29:60908–60921**

10.1007/s11356-022-20089-1

The publication of this article was funded by the Deutsche Forschungsgemeinschaft (DFG, German Research Foundation) – project number 491292795.

