# Supplementary material for: Spatial variation of rodenticides and emerging contaminants in blood of raptor nestlings from Germany
Source: Environ Sci Pollut Res Int. 2022 Apr 18;29(40):60908–21. doi: 10.1007/s11356-022-20089-1 (PMC9427910; doi:10.1007/s11356-022-20089-1)
Supplement: Supplementary file 1 — Supplementary file1 (PDF 1477 KB) [file 11356_2022_20089_MOESM1_ESM.pdf]

Supplementary information for

Spatial variation of rodenticides and emerging contaminants in blood of raptor nestlings from Germany

Alexander Badry[a*], Detlef Schenke[b], Helmut Brücher[c], Nayden Chakarov[d], Thomas Grünkorn[e], Hubertus Illner[f], Oliver Krüger[d], Torsten Marczak[g], Gerard Müskens[h], Winfried Nachtigall[i], Ronald Zollinger[j], Gabriele Treu[k], Oliver Krone[a]

[a] Leibniz Institute for Zoo and Wildlife Research, Department of Wildlife Diseases, Alfred-Kowalke-Straße 17, 10315 Berlin, Germany

[b] Julius Kühn-Institut, Institute for Ecological Chemistry, Plant Analysis and Stored Product Protection, Königin-Luise-Straße 19, 14195 Berlin, Germany

[c] Wiesenweihenschutz Brandenburg, Hauptstraße 11, 14913 Rohrbeck, Germany

[d] Department of Animal Behaviour, Bielefeld University, Morgenbreede 45, 33615 Bielefeld, Germany

[e] BioConsult SH, Schobüller Straße 36, 25813 Husum, Germany

[f] Arbeitsgemeinschaft Biologischer Umweltschutz / Biologische Station Soest, Teichstraße 19, 59505 Bad Sassendorf

[g] Independent, Germany

[h] Müskens Fauna, van Nispenstraat 4, 6561 BG Groesbeek, The Netherlands

[i] Förderverein Vogelschutzwarte Neschwitz, Park 4, 02699 Neschwitz, Germany

[j] Natuurplaza, PO Box 1413, NL-6501 BK Nijmegen, The Netherlands

[k] Umweltbundesamt, Department Chemicals, Wörlitzer Platz 1, 06844 Dessau-Roßlau, Germany

*Corresponding author's contact details:
Alexander Badry: a.badry@outlook.de

Table of contents

Tables SI-1-7 1

Figures SI-1-4 24

References 28

Tables SI-1-7

Table SI-1: Biometric data of the investigated species. Common buzzards (*Buteo buteo*, BUBT), Montagu's harriers (*Circus pygargus*, CIPY), white-tailed sea eagles (*Haliaeetus albicilla*, HAAL), red kites (*Milvus milvus*, MIML), and ospreys (*Pandion haliaetus*, PAHA). NA = no data available

Id	Species	Year	Wing length	Weight	No of nestlings per nest
1	BUBT	2019	92	420	3
2	BUBT	2019	200	660	2
3	BUBT	2019	143	575	2
4	BUBT	2019	149	715	1
5	BUBT	2019	191	725	3
6	BUBT	2019	158	620	3
7	BUBT	2019	275	820	3
47	BUBT	2019	222	690	NA
48	BUBT	2019	235	940	1
49	BUBT	2019	232	880	2
84	BUBT	2020	243	840	1
85	BUBT	2020	241	925	3
86	BUBT	2020	211	835	1
87	BUBT	2020	317	920	2
102	BUBT	2020	246	745	2
103	BUBT	2020	197	830	2
104	BUBT	2020	173	860	2
105	BUBT	2020	159	530	3
106	BUBT	2020	129	600	2
107	BUBT	2020	146	645	1
108	BUBT	2020	187	695	3
109	BUBT	2020	183	700	1
110	BUBT	2020	279	815	2
111	BUBT	2020	184	680	3
112	BUBT	2020	151	660	2
122	BUBT	2020	244	870	1
128	BUBT	2020	NA	NA	NA

129	BUBT	2020	NA	NA	1
130	BUBT	2020	NA	NA	1
131	BUBT	2020	NA	NA	3
132	BUBT	2020	NA	NA	2
133	BUBT	2020	NA	NA	2
134	BUBT	2020	NA	NA	2
135	BUBT	2020	NA	NA	2
161	BUBT	2020	112	560	NA
32	CIPY	2019	230	335	7
33	CIPY	2019	282	337	3
34	CIPY	2019	289	333	3
35	CIPY	2019	218	388	5
36	CIPY	2019	266	378	1
37	CIPY	2019	216	375	4
38	CIPY	2019	161	315	3
39	CIPY	2019	243	372	4
40	CIPY	2019	266	358	3
41	CIPY	2019	236	308	4
42	CIPY	2019	191	343	2
43	CIPY	2019	249	314	3
44	CIPY	2019	192	336	4
45	CIPY	2019	230	327	4
46	CIPY	2019	221	262	2
88	CIPY	2020	189	340	4
89	CIPY	2020	200	330	5
90	CIPY	2020	266	310	4
91	CIPY	2020	189	330	4
92	CIPY	2020	280	320	4
93	CIPY	2020	230	398	4
94	CIPY	2020	228	315	4
95	CIPY	2020	188	387	3
96	CIPY	2020	229	358	3
97	CIPY	2020	204	393	5

98	CIPY	2020	254	336	4
99	CIPY	2020	251	337	5
100	CIPY	2020	274	310	3
101	CIPY	2020	191	400	2
136	HAAL	2019	NA	NA	NA
137	HAAL	2019	NA	NA	1
138	HAAL	2019	NA	NA	1
139	HAAL	2019	NA	NA	2
140	HAAL	2019	NA	NA	1
141	HAAL	2019	NA	NA	1
142	HAAL	2019	NA	NA	2
143	HAAL	2019	NA	NA	1
144	HAAL	2019	NA	NA	3
145	HAAL	2019	NA	NA	1
146	HAAL	2019	NA	NA	3
147	HAAL	2019	NA	NA	1
148	HAAL	2019	NA	NA	2
149	HAAL	2019	NA	NA	2
162	HAAL	2019	292	3920	2
163	HAAL	2019	343	3810	2
164	HAAL	2019	318	3880	1
165	HAAL	2019	232	3610	2
166	HAAL	2019	336	3330	1
167	HAAL	2019	325	3870	1
168	HAAL	2019	178	2300	1
169	HAAL	2019	211	2890	2
170	HAAL	2019	303	3970	1
171	HAAL	2019	159	2630	1
172	HAAL	2019	396	5320	2
173	HAAL	2019	312	4400	2
174	HAAL	2019	331	4090	2
175	HAAL	2019	345	4500	2
176	HAAL	2019	436	4220	2

177	HAAL	2019	502	5450	2
178	HAAL	2019	451	4310	2
179	HAAL	2019	489	5620	2
180	HAAL	2019	406	3780	2
181	HAAL	2019	484	3990	2
150	HAAL	2020	NA	NA	2
151	HAAL	2020	NA	NA	2
152	HAAL	2020	NA	NA	2
153	HAAL	2020	NA	NA	2
154	HAAL	2020	NA	NA	1
155	HAAL	2020	NA	NA	2
156	HAAL	2020	NA	NA	2
157	HAAL	2020	NA	NA	1
158	HAAL	2020	NA	NA	1
159	HAAL	2020	NA	NA	1
160	HAAL	2020	NA	NA	2
182	HAAL	2020	352	5060	2
183	HAAL	2020	213	3540	1
184	HAAL	2020	233	3150	2
185	HAAL	2020	415	3510	1
186	HAAL	2020	273	3310	2
187	HAAL	2020	271	3800	2
188	HAAL	2020	407	5100	1
189	HAAL	2020	352	4650	1
190	HAAL	2020	497	5170	1
191	HAAL	2020	336	3610	1
192	HAAL	2020	346	3670	1
193	HAAL	2020	296	4340	1
194	HAAL	2020	383	4430	2
195	HAAL	2020	514	5360	1
196	HAAL	2020	355	3820	2
197	HAAL	2020	381	3880	1
198	HAAL	2020	285	4290	1

199	HAAL	2020	245	3670	1
200	HAAL	2020	254	3540	2
8	MIML	2019	259	920	2
9	MIML	2019	217	630	1
10	MIML	2019	204	660	2
11	MIML	2019	287	880	1
12	MIML	2019	255	900	1
13	MIML	2019	254	860	3
14	MIML	2019	184	720	2
15	MIML	2019	240	810	3
16	MIML	2019	242	790	2
17	MIML	2019	317	950	2
18	MIML	2019	305	990	2
19	MIML	2019	170	630	2
20	MIML	2019	350	910	2
21	MIML	2019	304	910	1
22	MIML	2019	338	1100	2
23	MIML	2019	356	1250	3
50	MIML	2019	335	1035	NA
51	MIML	2019	203	840	3
52	MIML	2019	368	1020	NA
53	MIML	2019	194	780	NA
54	MIML	2019	258	900	1
55	MIML	2019	350	1040	2
56	MIML	2019	206	840	NA
57	MIML	2019	199	895	NA
58	MIML	2020	263	870	1
59	MIML	2020	329	1010	3
60	MIML	2020	285	940	1
61	MIML	2020	296	870	3
62	MIML	2020	334	910	2
63	MIML	2020	218	880	1
64	MIML	2020	295	980	2

65	MIML	2020	325	1010	3
66	MIML	2020	331	950	2
67	MIML	2020	250	790	2
68	MIML	2020	301	890	2
69	MIML	2020	359	895	3
70	MIML	2020	345	1020	3
71	MIML	2020	340	1000	1
72	MIML	2020	323	920	3
73	MIML	2020	353	950	2
74	MIML	2020	356	885	3
75	MIML	2020	351	1000	NA
76	MIML	2020	330	1045	2
113	MIML	2020	323	990	3
114	MIML	2020	269	905	3
115	MIML	2020	241	905	2
116	MIML	2020	192	815	1
117	MIML	2020	319	NA	1
118	MIML	2020	344	1030	3
119	MIML	2020	35	970	3
120	MIML	2020	281	1010	2
121	MIML	2020	152	710	2
123	MIML	2020	284	1050	3
24	PAHA	2019	315	1250	2
25	PAHA	2019	320	1150	2
26	PAHA	2019	289	1150	3
27	PAHA	2019	271	1040	3
28	PAHA	2019	303	1460	3
29	PAHA	2019	338	1600	3
30	PAHA	2019	323	1230	3
31	PAHA	2019	330	1700	4
201	PAHA	2019	308	1720	3
202	PAHA	2019	218	1270	3
203	PAHA	2019	245	1290	2

204	PAHA	2019	361	1370	2
205	PAHA	2019	364	1500	1
77	PAHA	2020	225	1340	1
78	PAHA	2020	204	820	2
79	PAHA	2020	352	1280	2
80	PAHA	2020	208	1000	2
81	PAHA	2020	213	1080	2
82	PAHA	2020	251	1400	2
83	PAHA	2020	226	1030	3
206	PAHA	2020	352	1460 (wet)	2
207	PAHA	2020	NA	1830 (wet)	2
208	PAHA	2020	364	1660	2

Table SI-2: Tonnages of plant protection products based on the inland sales (Germany) of active substances in 2019 (BVL, 2020). Dates of withdrawn plant protection products during the sampling period (until 01/08/2021) are indicated in bold. Further information can be found here: https://ec.europa.eu/food/plant/pesticides/eu-pesticides-database/active-substances/?event=search.as. Sales of veterinary medicinal products in 2019 were based on Wallmann et al. (2020). HMP: Human Medicinal Product. VMP: Veterinary Medicinal Product.

Name	Intended use	CAS	Detected	End of Authorisation	Tonnage (2019)
Brodifacoum	Biocide	56073-10-0			
Bromadiolone	Biocide	28772-56-7			
Chlorophacinone	Biocide	3691-35-8			
Coumatetralyl	Biocide	5836-29-3			
Difenacoum	Biocide	56073-07-5			
Difethialone	Biocide	104653-34-1			
Flocoumafen	Biocide	90035-08-8			
Warfarin	Biocide	81-81-2			
2,4-D	Herbicide	94-75-7		31/12/2030	25-100
Acetamiprid	Insecticide	135410-20-7		28/02/2033	10-25
Aclonifen	Herbicide	74070-46-5		31/07/2022	250-1000
Amisulbrom	Fungicide	348635-87-0		30/09/2024	<1
Azoxystrobin	Fungicide	131860-33-8		31/12/2024	250-1000
Bentazone	Herbicide	25057-89-0		31/05/2025	/
Bixafen	Fungicide	581809-46-3		31/05/2025	25-100
Boscalid	Fungicide	188425-85-6		31/07/2022	100-250
Bromoxynil	Herbicide	1689-84-5	X	**31/07/2021**	25-100
Chlorantraniliprole	Insecticide	500008-45-7		31/12/2024	2.5-10
Chloridazon	Herbicide	1698-60-8		**31/12/2018**	1.0-2.5
Chlorotoluron	Herbicide	15545-48-9		31/10/2021	250-1000
Chlorpyrifos	Insecticide	2921-88-2		**16/01/2020**	/
Clothianidin	Insecticide	210880-92-5		**31/01/2019**	/export
Cyazofamid	Fungicide	120116-88-3		31/07/2036	10-25
Cyprodinil	Fungicide	121552-61-2		30/04/2022	25-100
Dichlorprop-P	Herbicide	15165-67-0		30/04/2022	25-100
Difenoconazole	Fungicide	119446-68-3		31/12/2021	100-250
Diflufenican	Herbicide	83164-33-4		31/12/2021	250-1000
Dimethachlor	Herbicide	50563-36-5		31/12/2021	10-25
Dimethenamid-P	Herbicide	163515-14-8		31/08/2034	250-1000
Dimethoate	Insecticide	60-51-5		**31/07/2019**	100-250
Dimethomorph	Fungicide	110488-70-5		31/07/2022	25-100
Dimoxystrobin	Fungicide	149961-52-4		31/01/2022	10-25
Epoxiconazole	Fungicide	106325-08-0		**30/04/2020**	100-250

Ethofumesate	Herbicide	26225-79-6		31/10/2031	250-1000
Famoxadone	Fungicide	131807-57-3		30/06/2022	2.5-10
Fenpropidin	Fungicide	67306-00-7	X	31/12/2021	100-250
Fenpropimorph	Fungicide	67564-91-4	X	**30/04/2019**	100-250
Fipronil	Insecticide/Biocide	120068-37-3		**30/09/2017**	/
Florasulam	Herbicide	145701-23-1		31/12/2030	10-25
Fludioxonil	Fungicide	131341-86-1		31/10/2021	25-100
Flufenacet	Herbicide	142459-58-3		31/10/2021	250-1000
Flumioxazin	Herbicide	103361-09-7		30/06/2022	2.5-10
Fluopicolide	Fungicide	239110-15-7		31/05/2023	10-25
Flupyrsulfuron-methyl	Herbicide	144740-53-4		**31/12/2017**	/
Fluroxypyr	Herbicide	69377-81-7		31/12/2024	100-250
Flurtamone	Herbicide	96525-23-4		**31/10/2019**	25-100
Fluxapyroxad	Fungicide	907204-31-3		31/05/2025	25-100
Foramsulfuron	Herbicide	173159-57-4		31/05/2035	25-100
Imazosulfuron	Herbicide	122548-33-8		**31/07/2017**	/
Imidacloprid	Insecticide	138261-41-3		01/12/2020	<1
Iodosulfuron-methyl	Herbicide	144550-06-1		31/03/2032	2.5-10
Isoproturon	Herbicide	34123-59-6		**31/12/2015**	/
Isopyrazam	Fungicide	881685-58-1		31/03/2023	25-100
Lenacil	Herbicide	2164-08-1		31/12/2021	25-100
MCPA	Herbicide	94-74-6	X	31/10/2021	250-1000
Mecoprop-P	Herbicide	16484-77-8		31/01/2021	25-100
Mesosulfuron-methyl	Herbicide	208465-21-8		30/06/2032	2.5-10
Mesotrione	Herbicide	104206-82-8		31/05/2032	100-250
Metamitron	Herbicide	41394-05-2		31/08/2022	1000-2500
Metazachlor	Herbicide	67129-08-2		31/07/2022	250-1000
Metconazole	Fungicide	125116-23-6		30/04/2022	25-100
Methiocarb	Insecticide	2032-65-7		**03/10/2019**	25-100
Metosulam	Herbicide	139528-85-1		30/04/2021	<1
Metrafenone	Fungicide	220899-03-6		07/04/2022	25-100
Metribuzin	Herbicide	21087-64-9		31/07/2022	25-100
Metsulfuron-methyl	Herbicide	74223-64-6		31/03/2023	2.5-10
Napropamide	Herbicide	15299-99-7		31/12/2023	25-100
Nicosulfuron	Herbicide	111991-09-4		31/12/2021	10-25
Omethoate	Dimethoate metab.	1113-02-6			/

Pendimethalin	Herbicide	40487-42-1		30/11/2024	250-1000
Pethoxamid	Herbicide	106700-29-2		30/11/2033	100-250
Picolinafen	Herbicide	137641-05-5		30/06/2031	1.0-2.5
Picoxystrobin	Fungicide	117428-22-5		**31/10/2017**	/
Pirimicarb	Insecticide	23103-98-2		30/04/2022	25-100
Prochloraz	Fungicide	67747-09-5		31/12/2023	100-250
Propiconazole	Fungicide	60207-90-1		19/12/2018	25-100
Propyzamide	Herbicide	23950-58-5		30/06/2025	100-250
Proquinazid	Fungicide	189278-12-4		31/07/2022	2.5-10
Prosulfuron	Herbicide	94125-34-5		31/07/2024	1.0-2.5
Pymetrozine	Insecticide	123312-89-0		**30/04/2019**	10-25
Pyraclostrobin	Fungicide	175013-18-0		31/01/2022	25-100
Pyroxsulam	Herbicide	422556-08-9		30/04/2025	10-25
Quinmerac	Herbicide	90717-03-6		31/07/2024	25-100
Quinoxyfen	Fungicide	124495-18-7		**30/04/2019**	2.5-10
S-Metolachlor	Herbicide	87392-12-9		31/07/2022	250-1000
Spinosyn A	Insecticide	131929-60-7		30/04/2022 (Spinosad)	2.5-10 (Spinosad)
Spiroxamine	Fungicide	118134-30-8	X	31/12/2023	250-1000
Sulcotrione	Fungicide	99105-77-8		31/08/2022	/
Tebuconazole	Fungicide	107534-96-3		31/08/2022	250-1000
Terbuthylazine	Herbicide	5915-41-3	X	31/12/2024	250-1000
Thiacloprid	Insecticide	111988-49-9		**03/02/2020**	25-100
Thiamethoxam	Insecticide	153719-23-4		**30/04/2019**	/
Thifensulfuron-methyl	Herbicide	79277-27-3		31/10/2031	10-25
Triadimenol	Fungicide	55219-65-3		**31/08/2019**	25-100
Triasulfuron	Herbicide	82097-50-5		**31/12/2015**	/
Trifloxystrobin	Fungicide	141517-21-7		31/07/2033	10-25
Tritosulfuron	Herbicide	142469-14-5		30/11/2021	10-25
Zoxamide	Fungicide	156052-68-5		30/06/2033	2.5-10
Ciprofloxacin	HMP	85721-33-1			
Diclofenac	HMP	15307-86-5			
Ibuprofen	HMP	15687-27-1			
Sulfadiazine	HMP	68-35-9			
Enrofloxacin	VMP	93106-60-6			4,770
Marbofloxacin	VMP	115550-35-1			1,155
Sulfamethazine	VMP	57-68-1			/

Table SI-3: Sample preparation and extraction.

Defrosting of blood samples, pipetting 200 µl in pp - tubes (15 mL)

⇩

Spike with surrogates (20 µl with 1 ng/µL)

⇩

Addition of 1.98 mL acetonitrile

⇩

Vortex with steel ball (30 sec)

⇩

Ultrasound 5 min

⇩

Centrifugation (Heraeus Megafuge 16 R) 10 min with 5,000 rpm at RT

⇩

Transfer an aliquot of supernatant in a new pp - tube

⇩

Repeat extraction with 2 mL acetonitrile

⇩

Combine the supernatants

⇩

Reduce aliquots (200 µL) to dryness with nitrogen

⇩

Resuspend the residue with 0.5 ml internal standards
in methanol/water for Method A, B, C, E
in acetonitrile/water for Method D

⇩

Briefly ultrasound

⇩

Filtration through syringe filter (0.2 µm PTFE)

⇩

Storage at -20°C until analysis

⇩

LC-MS/MS

Table SI-4: Configuration of LC-MS/MS for five methods (A-E).

Method A

**LIQUID CHROMATOGRAPHY**	**Agilent Infinity 1290 II**		
Autosampler temperature	10 °C		
Injection volume	10 µL		
Analytical column	Agilent Zorbax Eclipse C18 (1.8 µm, 50 mm, 2.1 mm i.d.)		
Column temperature	40 °C		
Mobile phase A	H_2O +1 mmol NH_4F		
Mobile phase B	Methanol / Acetonitrile (65/35)		
Gradient program	Time (min)	A (%)	B (%)
	0.0	98	2
	2.5	2	98
	4.0	2	98
	4.1	98	2
	6.0	98	2
Flow rate	500 µL/min		
**MASS SPECTROMETER**	**QTRAP 6500+ (SCIEX)**		
Mode	negative ESI		
Ion spray potential	-4500 V		
Source temperature	550 °C		
Scan type	Multiple Reaction Monitoring / Enhanced Product Ion		

Method B

**LIQUID CHROMATOGRAPHY**	**Agilent Infinity 1290 II**		
Autosampler temperature	10 °C		
Injection volume	5 µL		
Analytical column	Agilent Zorbax Eclipse C18 (1,8 µm, 50 mm, 2.1 mm i.d.)		
Column temperature	40 °C		
Mobile phase A	H_2O +5 mmol NH_4formate + 0.5% formic acid		
Mobile phase B	Methanol +5 mmol NH_4formate +0.5% formic acid		
Gradient program	Time (min)	A (%)	B (%)
	0.0	98	2
	2.5	2	98
	5.0	2	98
	5.1	98	2
	6.0	98	2
Flow rate	500 µL/min		
**MASS SPECTROMETER**	**QTRAP 6500+ (SCIEX)**		
Mode	positive ESI		
Ion spray potential	5500 V		
Source temperature	550 °C		
Scan type	Multiple Reaction Monitoring / Enhanced Product Ion		

Method C

LIQUID CHROMATOGRAPHY	Agilent Infinity 1290 II		
Autosampler temperature	10 °C		
Injection volume	5 µL		
Analytical column	Agilent Zorbax Eclipse C18 (1.8 µm, 50 mm, 2.1 mm i.d.)		
Column temperature	40 °C		
Mobile phase A	H_2O +1 mmol NH_4F		
Mobile phase B	Methanol / Acetonitrile (65/35)		
Gradient program	Time (min)	A (%)	B (%)
	0.00	98	2
	3.00	2	98
	4.50	2	98
	4.51	98	2
	6.00	98	2
Flow rate	500 µL/min		
**MASS SPECTROMETER**	**QTRAP 6500 (SCIEX)**		
Mode	positive ESI		
Ion spray potential	5500 V		
Source temperature	550 °C		
Scan type	Multiple Reaction Monitoring / Enhanced Product Ion		

Method D

LIQUID CHROMATOGRAPHY	Agilent Infinity 1290 II		
Autosampler temperature	10 °C		
Injection volume	10 µL		
Analytical column	Agilent Zorbax Eclipse (1.8 µm, 50 mm, 2.1 mm i.d.)		
Column temperature	40 °C		
Mobile phase A	H_2O +1 mmol NH_4F + 0.1% formic acid		
Mobile phase B	Acetonitrile + 0.1% formic acid		
Gradient program	Time (min)	A (%)	B (%)
	0.0	98	2
	0.5	98	2
	3.0	2	98
	4.0	2	98
	4.1	98	2
	5.0	98	2
Flow rate	500 µL/min		
**MASS SPECTROMETER**	**QTRAP 6500+ (SCIEX)**		
Mode	positive ESI		
Ion spray potential	5500 V		
Source temperature	500 °C		
Scan type	Multiple Reaction Monitoring / Enhanced Product Ion		

Method E

LIQUID CHROMATOGRAPHY	Agilent Infinity 1290 II		
Autosampler temperature	10 °C		
Injection volume	10 µL		
Analytical column	Agilent Zorbax Eclipse C18 (1.8 µm, 50 mm, 2.1 mm i.d.)		
Column temperature	40 °C		
Mobile phase A	H_2O +1 mmol NH_4F		
Mobile phase B	Methanol / Acetonitrile (65/35)		
Gradient program	Time (min)	A (%)	B (%)
	0.0	98	2
	2.5	2	98
	4.0	2	98
	4.1	98	2
	5.0	98	2
Flow rate	500 µL/min		
**MASS SPECTROMETER**	**QTRAP 6500+ (SCIEX)**		
Mode	negative ESI		
Ion spray potential	-4500 V		
Source temperature	500 °C		
Scan type	Multiple Reaction Monitoring / Enhanced Product Ion		

Table SI-5: LC-MS/MS – MRM- and EPI-conditions (precursor (Q1) and product ions (Q3) in m/z and declustering potential (DP), entrance potential (EP), collision energy (CE) and cell exit potential (CXP) in V).

Q1	Q3	Analyte	DP	EP	CE	CXP
		**Method A**				
520.9	78.8	Brodifacoum	-20	-10	-128	-11
526.9	249.9	Bromadiolone	-30	-10	-50.	-19
373.1	201.0	Chlorophacinone	-75	-10	-30	-13
291.0	140.9	Coumatetralyl	-125	-10	-38	-13
443.1	135.0	Difenacoum	-55	-10	-46	-15
538.9	80.8	Difethialone	-20	-10	-92	-13
541.0	382.0	Flocoumafen	-65	-10	-36	-29
307.0	161.0	Warfarin	-45	-10	-26	-19
351.9	265.0	Acenocoumarol (Surr)	-70	-10	-40	-13
532.0	255	Bromadiolone D5 (Surr)	-30	-10	-50	-19
340.9	160.8	Coumachlor (Surr)	-60	-10	-30	-21
343.1	167.0	Diphacinone D4 (Surr)	-115	-10	-32	-15
278.9	250.0	Phenprocoumon (Surr)	-55	-10	-32	-17
377.1	200.9	Chlorophacinone D4 (IS)	-120	-10	-32	-15
312.1	161.0	Warfarin D5 (IS)	-95	-10	-28	-9
		**Method B**				
223.1	125.9	Acetamiprid	101	10	33	8
265.0	182.1	Aclonifen	36	10	39	2.6
403.9	372.0	Azoxystrobin	51	10	17	24
414.1	394.0	Bixafen	76	10	21	24
343.0	307.0	Boscalid	54	10	27	3.6
222.0	92.2	Chloridazon	39	10	35	1.8
213.1	72.0	Chlorotoluron	34	10	33	1.6
350.0	96.7	Chlorpyrifos	51	10	55	11
250.1	169.0	Clothianidin	42	10	19	12

226.1	108.0	Cyprodinil	106	10	35	8
406.1	250.9	Difenoconazole	41	10	37	3.3
395.1	266.1	Diflufenican	81	10	35	22
256.1	224.2	Dimethachlor	24	10	19	3
276.1	244.1	Dimethenamid-P	86	10	19	12
230.0	125.0	Dimethoate	14	10	29	2.1
388.1	301.1	Dimethomorph	41	10	27	3.6
329.9	121.0	Epoxiconazole	66	10	29	12
392.0	331.0	Famoxadone	26	10	13	15
274.2	147.1	Fenpropidin	31	10	37	2.3
304.3	147.1	Fenpropimorph	34	10	39	2.3
360.0	128.9	Florasulam	21	10	31	16
383.1	172.8	Fluopicolide	31	10	27	20
466.0	182.0	Flupyrsulfuron-methyl	91	10	29	10
255.0	209.1	Fluroxypyr	49	10	21	2.9
334.2	247.2	Flurtamone	86	10	35	20
381.8	342.0	Fluxapyroxad	50	10	30	10
453.1	182.2	Foramsulfuron	31	10	27	10
413.0	156.0	Imazosulfuron	25	10	20	20
256.1	175.0	Imidacloprid	49	10	25	10
507.8	167.0	Iodosulfuron-methyl	71	10	25	10
207.1	72.0	Isoproturon	46	10	19	10
360.2	244.0	Isopyrazam	56	10	31	22
235.1	153.1	Lenacil	34	10	21	10
504.0	182.0	Mesosulfuron-methyl	81	10	31	10
203.1	175.0	Metamitron	49	10	29	2.5
278.1	210.1	Metazachlor	15	10	15	20
417.9	175.0	Metosulam	61	10	35	14
215.1	187.2	Metribuzin	29	10	25	2.6
382.1	198.9	Metsulfuron-methyl	34	10	27	10
272.1	129.1	Napropamid	46	10	21	10
282.0	212.0	Pendimethalin	50	10	15	25
296.1	131.1	Pethoxamid	39	10	27	2.1
377.1	237.9	Picolinafen	46	10	39	14
239.2	182.3	Pirimicarb	66	10	21	10
342.1	69.1	Propiconazole	15	10	25	15
256.0	173.1	Propyzamide	39	10	31	2.5
372.9	331.0	Proquinazid	66	10	19	24
420.0	141.0	Prosulfuron	76	10	27	16
218.1	104.9	Pymetrozine	61	10	29	12
435.1	194.9	Pyroxsulam	51	10	35	18
222.0	204.1	Quinmerac	24	10	23	2.8
307.9	162.0	Quinoxyfen	21	10	57	2.4
284.1	251.9	S-Metolachlor	14	10	19	3.3
732.4	142.1	Spinosyn A	81	10	39	10
298.3	144.2	Spiroxamine	41	10	27	2.3
346.0	139.0	Sulcotrione	41	10	31	2.3
308.1	70.0	Tebuconazole	86	10	51	10
230.2	174.0	Terbuthylazine	106	10	23	54

252.8	126.0	Thiacloprid	41	10	33	8
388.0	167.0	Thifensulfuron-methyl	29	10	21	10
402.1	167.1	Triasulfuron	44	10	25	2.5
409.1	186.1	Trifloxystrobin	19	10	23	10
446.0	195.0	Tritosulfuron	50	10	30	10
336.0	187.0	Zoxamide	36	10	31	16
253.0	172.0	Clothianidin D3 (Surr)	42	10	19	14
412.1	250.9	Difenoconazole D6 (Surr)	41	10	37	3.3
236.0	131.0	Dimethoate D6 (Surr)	14	10	29	10
235.0	179.0	Terbuthylazin D5 (Surr)	76	10	25	10
257.1	126.0	Thiacloprid D4 (Surr)	116	10	31	14
226.0	126.0	Acetamiprid D3 (IS)	56	10	31	10
221.1	179.1	Atrazin D5 (IS)	21	10	25	10
295.0	70.0	Cyproconazol D3 (IS)	16	10	35	10
260.0	213.0	Imidacloprid D4 (IS)	86	10	23	10
213.2	78.0	Isoproturon D6 (IS)	1	10	21	6
147.9	97.0	Methamidiohos D6 (IS)	56	10	23	10
229.2	168.9	Methiocarb D3 (IS)	15	10	15	25
**Method C**						
466.0	226.9	Amisulbrom	15	10	30	25
484.2	452.9	Chlorantraniliprole	91	10	23	10
325.1	107.9	Cyazofamid	15	10	20	15
327.2	205.1	Dimoxystrobin	49	10	15	10
304.0	240.8	Ethofumesate	15	10	20	20
364.0	194.1	Flufenacet	21	10	15	12
355.0	299.0	Flumioxazin	100	10	45	10
320.1	70.1	Metconazole	36	10	45	10
226.1	169.1	Methiocarb	15	10	15	20
409.1	209.1	Metrafenone	39	10	21	10
411.0	182.1	Nicosulfuron	41	10	25	10
214.1	109.0	Omethoate	31	11.5	35	10
282.1	212.0	Pendimethalin	1	10	15	10
368.1	145.0	Picoxystrobin	39	10	27	10
375.9	308.0	Prochloraz	25	10	20	15
189.2	102.0	Propamocarb	16	10	23	1.9
388.1	194.0	Pyraclostrobin	19	10	19	10
251.3	155.9	Sulfadiazin	106	10	21	10
279.3	186.0	Sulfamethazin	56	10	27	10
292.0	211.0	Thiamethoxam	34	10	17	10
296.1	69.9	Triadimenol	46	10	33	10
255.1	160.1	Sulfdiazin D4 (Surr)	71	10	23	10
221.1	179.1	Atrazin D5 (IS)	21	10	25	10
295.0	70.0	Cyproconazol D3 (IS)	16	10	35	10
147.9	97.0	Methamidiohos D6 (IS)	56	10	23	10
229.2	168.9	Methiocarb D3 (IS)	15	10	15	25
**Method D**						
332.0	314.0	Ciprofloxacin	1	10	29	16
360.0	342.1	Enrofloxacin	1	10	29	18
363.0	345.0	Marbofloxacin	76	10	29	18

339.9	322.1	Ciprofloxacin D8 (Surr)	121	10	29	18
221.1	179.1	Atrazine D5 (IS)	21	10	25	10
**Method E**						
218.9	160.9	2.4 D	-45	-10	-16	-13
239.0	132.0	Bentazone	-55	-10	-36	-9
275.7	81.0	Bromoxynil	-60	-10	-62	-7
232.9	160.8	Dichlorprop-P	-50	-10	-18	-15
293.9	250.0	Diclofenac	-55	-10	-10	-16
373.0	282.0	Famoxadone	-65	-10	-26	-25
434.9	329.9	Fipronil	-5	-10	-22	-19
247.0	180.0	Fludioxonil	-35	-10	-40	-9
205.0	161.0	Ibuprofen	-45	-10	-10	-9
199.0	140.9	MCPA	-60	-10	-20	-9
213.0	140.9	Mecoprop-P	-65	-10	-20	-17
338.0	291.0	Mesotrione	-20	-10	-20	-10
327.0	291.0	Sulcotrione	-20	-10	-10	-20
298.1	254.0	Diclofenac D4 (Surr)	-55	-10	-16	-19
312.1	161.0	Warfarin D5 (IS)	-95	-10	-28	-9

**EPI (enhanced product ion spectra)**				
**Mass range**		**DP**	**EP**	**CE**
50 – 450 m/z		-50/+50	-10/+10	-30/+30 (± 15)
EPI spectra in the sample agree > 80% with standards in the same sequence (response >500 cps)				

Software	Analyst 1.7.1
Quantification	Relative peak area

Matrix matched standard: 0.01 - 20 pg/µl

Table SI-6a: Validation - RECovery of analytes (10, 100, 1000 ng/ml pig blood; n =5 / in control samples all not detected; n = 2 / (RSD= relative standard deviation) and Reporting Limit (RL) (n. d. (not detected) = < RL) (HMP=human medicinal product; VMP= veterinary medicinal product).

Analyte	Intended use	RL	REC	RSD	REC	RSD	REC	RSD
		ng/ml	10 ng/ml		100 ng/ml		1000 ng/ml	
			%		%		%	
Brodifacoum	Biocide	5	**71**	15	**84**	5	**83**	8
Bromadiolone	Biocide	5	**79**	9	**101**	8	**94**	6
Chlorophacinone	Biocide	10	**84**	8	**90**	4	**88**	4
Coumatetralyl	Biocide	0.5	**95**	4	**87**	2	**86**	5
Difenacoum	Biocide	2.5	**86**	13	**94**	6	**89**	7
Difethialone	Biocide	2.5	**66**	16	**88**	10	**84**	11
Flocoumafen	Biocide	0.5	**74**	17	**93**	8	**87**	9
Warfarin	Biocide	0.5	**105**	6	**102**	4	**101**	4
2,4-D	Herbicide	5	**77**	8	**77**	5	**72**	10
Acetamiprid	Insecticide	5	**96**	9	**91**	4	**94**	4
Aclonifen	Herbicide	25	**n. d.**		**65**	5	**75**	9
Amisulbrom	Fungicide	5	**70**	24	**67**	10	**84**	9
Azoxystrobin	Fungicide	1	**99**	7	**93**	7	**99**	11
Bentazone	Herbicide	0.5	**88**	5	**90**	4	**90**	3
Bixafen	Fungicide	1	**89**	8	**89**	4	**94**	3
Boscalid	Fungicide	2.5	**92**	9	**82**	4	**91**	5
Bromoxynil	Herbicide	2.5	**79**	7	**79**	4	**90**	3
Chlorantraniliprole	Insecticide	1	**88**	6	**87**	4	**90**	3
Chloridazon	Herbicide	25	**n. d.**		**91**	5	**90**	5
Chlorotoluron	Herbicide	2.5	**92**	5	**85**	4	**85**	5
Chlorpyrifos	Insecticide	5	**57**	4	**44**	22	**33**	21
Clothianidin	Insecticide	5	**88**	18	**90**	13	**90**	9
Cyazofamid	Fungicide	0.5	**78**	9	**75**	8	**78**	3
Cyprodinil	Fungicide	25	**n. d.**		**62**	10	**62**	6
Dichlorprop-P	Herbicide	0.5	**72**	9	**77**	6	**75**	7
Difenoconazole	Fungicide	2.5	**86**	6	**79**	10	**81**	4
Diflufenican	Herbicide	5	**85**	12	**76**	9	**76**	10
Dimethachlor	Herbicide	2.5	**70**	11	**55**	33	**37**	21
Dimethenamid-P	Herbicide	5	**56**	15	**43**	37	**28**	33
Dimethoate	Insecticide	5	**86**	7	**82**	13	**70**	10
Dimethomorph	Fungicide	2.5	**96**	6	**93**	4	**96**	4
Dimoxystrobin	Fungicide	1	**89**	7	**83**	6	**84**	2
Epoxiconazole	Fungicide	2.5	**87**	5	**83**	7	**87**	4
Ethofumesate	Herbicide	10	**80**	14	**63**	12	**67**	15
Famoxadone	Fungicide	2.5	**69**	30	**69**	17	**57**	28
Fenpropidin	Fungicide	2.5	**63**	9	**50**	23	**20**	31
Fenpropimorph	Fungicide	1	**55**	6	**45**	25	**23**	24
Fipronil	Insecticide/Biocide	0.5	**91**	8	**88**	7	**95**	5
Florasulam	Herbicide	5	**96**	5	**91**	4	**88**	3
Fludioxonil	Fungicide	0.5	**87**	8	**84**	4	**94**	3
Flufenacet	Herbicide	0.5	**81**	6	**72**	11	**68**	6

Flumioxazin	Herbicide	5	**n.d.**		**41**	14	**71**	14
Fluopicolide	Fungicide	5	**91**	6	**87**	6	**94**	4
Flupyrsulfuron-methyl	Herbicide	5	**90**	12	**89**	8	**92**	7
Fluroxypyr	Herbicide	50	**n.d.**		**56**	11	**82**	8
Flurtamone	Herbicide	2.5	**98**	15	**94**	7	**91**	9
Fluxapyroxad	Fungicide	2.5	**94**	8	**91**	3	**97**	4
Foramsulfuron	Herbicide	5	**88**	13	**85**	8	**82**	4
Imazosulfuron	Herbicide	2.5	**98**	12	**108**	12	**124**	9
Imidacloprid	Insecticide	5	**74**	12	**84**	6	**86**	4
Iodosulfuron-methyl	Herbicide	5	**102**	5	**97**	5	**95**	1
Isoproturon	Herbicide	2.5	**88**	7	**82**	6	**80**	5
Isopyrazam	Fungicide	2.5	**97**	7	**93**	4	**95**	3
Lenacil	Herbicide	25	**n.d.**		**87**	10	**96**	10
MCPA	Herbicide	1	**69**	5	**74**	5	**72**	11
Mecoprop-P	Herbicide	0.5	**70**	2	**75**	5	**71**	13
Mesosulfuron-methyl	Herbicide	2.5	**101**	13	**93**	5	**94**	8
Mesotrione	Herbicide	50	**n.d.**		**76**	14	**106**	13
Metamitron	Herbicide	25	**n.d.**		**85**	13	**81**	6
Metazachlor	Herbicide	1	**90**	9	**81**	13	**70**	7
Metconazole	Fungicide	0.5	**94**	5	**90**	5	**92**	3
Methiocarb	Insecticide	0.5	**86**	7	**75**	12	**74**	7
Metosulam	Herbicide	5	**92**	3	**91**	6	**86**	3
Metrafenone	Fungicide	0.5	**90**	5	**80**	5	**86**	4
Metribuzin	Herbicide	5	**74**	9	**73**	18	**73**	9
Metsulfuron-methyl	Herbicide	5	**83**	13	**88**	6	**93**	6
Napropamide	Herbicide	5	**87**	5	**79**	10	**73**	7
Nicosulfuron	Herbicide	50	**n.d.**		**66**	19	**75**	6
Omethoate	Dimethoate metab.	2.5	**79**	15	**70**	14	**65**	16
Pendimethalin	Herbicide	5	**75**	9	**51**	26	**40**	7
Pethoxamid	Herbicide	1	**88**	11	**75**	12	**65**	9
Picolinafen	Herbicide	2.5	**86**	9	**75**	7	**82**	6
Picoxystrobin	Fungicide	10	**85**	17	**67**	19	**74**	17
Pirimicarb	Insecticide	2.5	**76**	7	**57**	28	**36**	19
Prochloraz	Fungicide	2.5	**83**	5	**78**	6	**84**	5
Propiconazole	Fungicide	5	**89**	9	**82**	8	**86**	5
Propyzamide	Herbicide	25	**n.d.**		**66**	12	**65**	11
Proquinazid	Fungicide	1	**80**	10	**64**	15	**61**	9
Prosulfuron	Herbicide	5	**96**	5	**94**	5	**101**	5
Pymetrozine	Insecticide	5	**95**	13	**88**	5	**85**	8
Pyraclostrobin	Fungicide	0.5	**87**	5	**80**	5	**87**	4
Pyroxsulam	Herbicide	2.5	**96**	10	**95**	5	**89**	6
Quinmerac	Herbicide	5	**58**	6	**62**	4	**72**	4
Quinoxyfen	Fungicide	5	**76**	14	**58**	10	**64**	7
S-Metolachlor	Herbicide	5	**77**	4	**60**	25	**45**	19
Spinosyn A	Insecticide	2.5	**95**	9	**90**	8	**88**	8
Spiroxamine	Fungicide	1	**58**	5	**44**	30	**20**	34

Sulcotrione	Fungicide	2.5	**84**	8	**85**	3	**84**	5
Tebuconazole	Fungicide	5	**89**	7	**84**	5	**90**	3
Terbuthylazine	Herbicide	5	**81**	5	**67**	15	**58**	12
Thiacloprid	Insecticide	2.5	**92**	7	**94**	13	**97**	3
Thiamethoxam	Insecticide	1	**97**	16	**88**	6	**89**	5
Thifensulfuron-methyl	Herbicide	5	**92**	10	**92**	5	**88**	8
Triadimenol	Fungicide	10	**59**	20	**87**	11	**100**	12
Triasulfuron	Herbicide	5	**96**	5	**96**	5	**89**	4
Trifloxystrobin	Fungicide	2.5	**103**	10	**89**	7	**90**	8
Tritosulfuron	Herbicide	5	**79**	19	**94**	8	**89**	7
Zoxamide	Fungicide	2.5	**92**	11	**85**	5	**85**	9
Ciprofloxacin	HMP	5	**62**	11	**76**	5	**96**	2
Diclofenac	HMP	1	**72**	10	**78**	5	**79**	4
Ibuprofen	HMP	5	**101**	35	**83**	8	**74**	7
Sulfadiazine	HMP	0.5	**85**	3	**85**	3	**88**	5
Enrofloxacin	VMP	2.5	**83**	5	**85**	4	**98**	4
Marbofloxacin	VMP	5	**65**	5	**81**	5	**96**	3
Sulfamethazine	VMP	0.5	**93**	5	**87**	5	**91**	2

Table SI-6b: Validation - RECovery of surrogates (100 ng/ml) added to samples of method development and in practice samples (RSD= relative standard deviation).

	Method		Practice	
**Surrogate**	**REC**	**RSD**	**REC**	**RSD**
	%			
**Method A**				
Acenocoumarol	**99**	7	**88**	3
Bromadiolone D5	**94**	8	**94**	6
Coumachlor	**86**	9	**91**	4
Diphacinone D4	**81**	23	**81**	4
Phenprocoumon	**92**	6	**85**	3
**Method B**				
Clothianidin D3	**99**	7	**94**	9
Difenoconazole D6	**96**	6	**85**	11
Dimethoate D6	**89**	12	**84**	17
Terbuthylazine D5	**90**	13	**68**	19
Thiacloprid D4	**98**	9	**92**	5
**Method C**				
Sulfdiazine D4	**79**	6	**84**	5
**Method D**				
Ciprofloxacin D8	**78**	29	**67**	5
**Method E**				
Diclofenac D4	**75**	7	**76**	4

Table SI-7: Median concentrations ($Q_{0.25}$-$Q_{0.75}$) in ng mL^{-1} for individuals with detectable residues and detection rate [%] of anticoagulant rodenticides (ARs), plant protection products (PPPs) and medicinal products (MPs) in blood of common buzzards (*Buteo buteo*, BUBT), red kites (*Milvus milvus*, MIML), Montagu's harrier (*Circus pygargus*, CIPY), white-tailed sea eagles (*Haliaeetus albicilla*, HAAL) and osprey (*Pandion haliaetus*, PAHA) from Germany. n. d. = not detected. n^+ = samples with detectable residues.

ng mL^{-1}		BUBT n=35	MIML n=53	CIPY n=29	HAAL n=64	PAHA n=23	Overall n=204
**Anticoagulant rodenticides (ARs)**	**Brodifacoum**	n.d.	13 (8-13)	n.d.	n.d.	n.d.	13 (8-13)
	Detection rate [%]	0	9.4	0	0	0	2.5
	**Coumatetralyl**	n.d.	1 (1-1.5)	n.d.	n.d.	n.d.	1 (1-1.5)
	Detection rate [%]	0	5.7	0	0	0	1.5
	**Difenacoum**	n.d.	6.5 (2.5-10.3)	n.d.	n.d.	n.d.	6.5 (2.5-10.3)
	Detection rate [%]	0	7.6	0	0	0	2.0
	**Difethialone**	27	n.d.	n.d.	n.d.	n.d.	27
	Detection rate [%]	2.9 (n^+=1)	0	0	0	0	0.5 (n^+=1)
	**Warfarin**	1 (1-1)	1	n.d.	n.d.	n.d.	1 (1-1)
	Detection rate [%]	5.7 (n^+=2)	1.9 (n^+=1)	0	0	0	1.5
**Plant protection products (PPPs)**	**Bromoxynil**	42 (29.5-154)	11.5 (9-25.8)	12 (12-16)	5	n.d.	15 (9-40)
	Detection rate [%]	20.0	22.6	31.0	1.6 (n^+=1)	0	14.2

	**Fenpropidin**	n.d.	n.d.	n.d.	6 (4.5-8.8)	n.d.	6 (4.5-8.75)
	Detection rate [%]	0	0	0	6.3	0	2.0
	**Fenpropimorph**	2 (2-2)	n.d.	n.d.	3	n.d.	2 (2-2.5)
	Detection rate [%]	5.7 ($n^+=2$)	0	0	1.6 ($n^+=1$)	0	1.5
	**MCPA**	n.d.	n.d.	1.5 (1.3-1.8)	n.d.	n.d.	1.5 (1.25-1.75)
	Detection rate [%]	0	0	6.9 ($n^+=2$)	0	0	1.0
	**Spiroxamine**	n.d.	n.d.	n.d.	3 (2.5-6)	n.d.	3 (2.5-6)
	Detection rate [%]	0	0	0	4.7	0	1.5
	**Terbuthylazine**	n.d.	n.d.	n.d.	4	n.d.	4
	Detection rate [%]	0	0	0	1.6 ($n^+=1$)	0	0.5 ($n^+=1$)
**Medicinal products (MPs)**	**Ciprofloxacin**	13 (12.5-13.5)	6 (6-6)	n.d.	n.d.	5.5 (5.3-5.8)	6 (6-12.5)
	Detection rate [%]	8.6	3.8 ($n^+=2$)	0	0	8.7 ($n^+=2$)	3.4

Figures SI-1-4

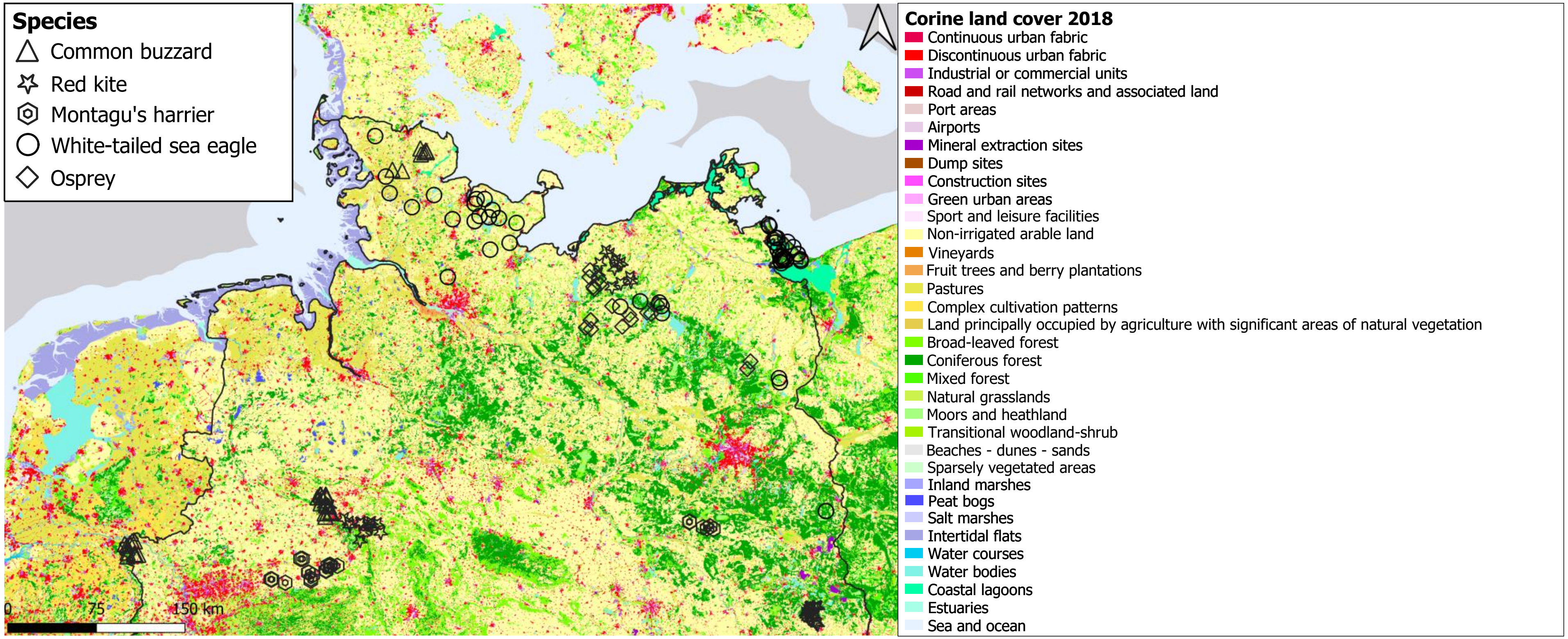

Figure SI-1: Land cover classes extracted from the Corine Land Cover 2018 (EEA, 2018) for the sampling area. Red coloured land cover classes approximate anthropogenic influences, yellow land cover classes approximate agricultural influences, green land cover classes approximate forest and semi-natural areas whereas blue land cover classes approximate aquatic areas. Sampling location of common buzzards (*Buteo buteo*, BUBT) are indicated by triangles, red kites (*Milvus milvus*, MIML) by stars, Montagu's harriers (*Circus pygargus*, CIPY) by doubled hexagons, white-tailed sea eagle (*Haliaeetus albicilla*, HAAL) by circles, and osprey (*Pandion haliaetus*, PAHA) by squares.

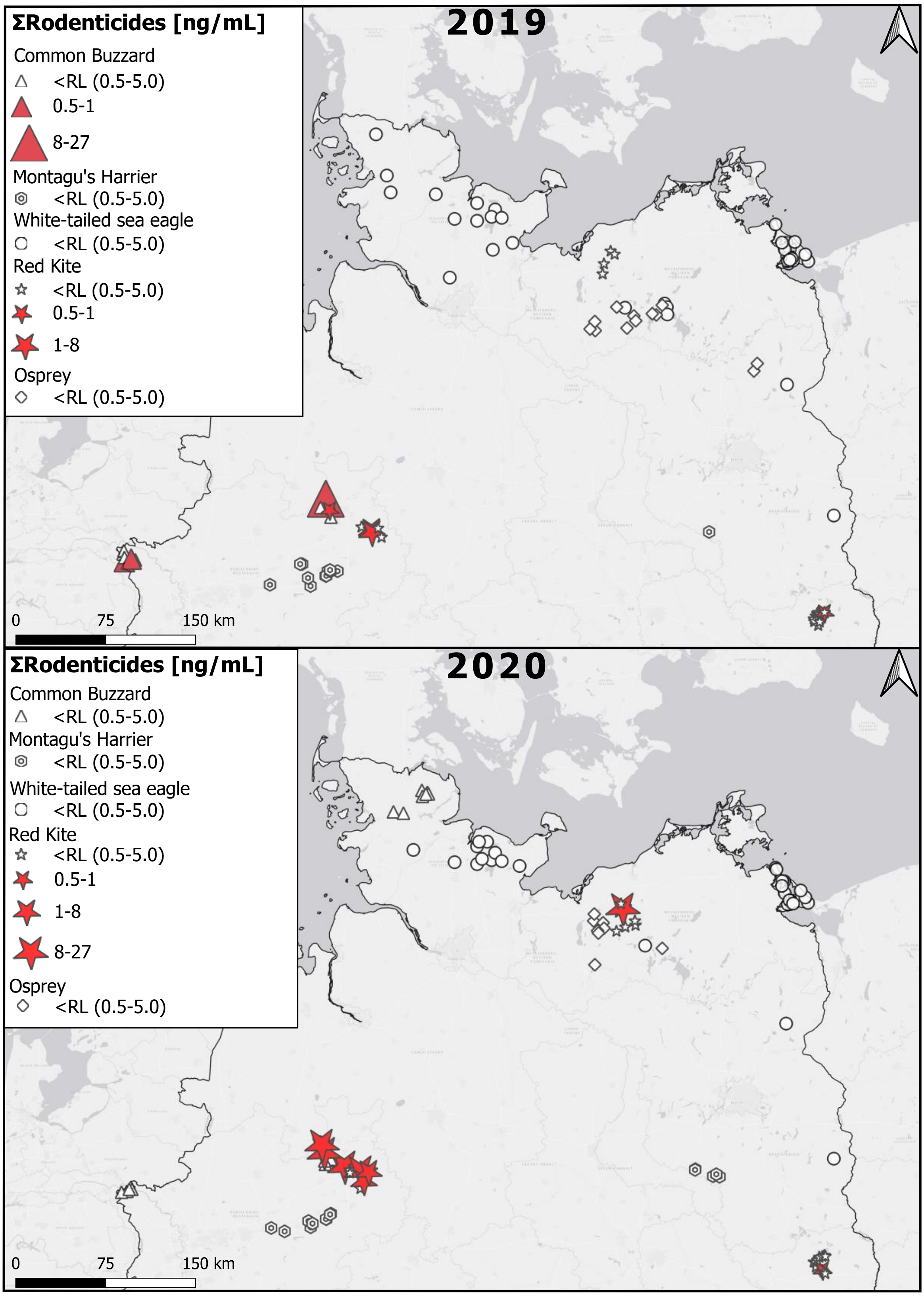

Figure SI-2: Spatial detection of ΣARs (rodenticides) (red) in blood of common buzzards (*Buteo buteo*, BUBT, triangles), Montagu's harriers (*Circus pygargus*, CIPY, doubled hexagons), white-tailed sea eagle (*Haliaeetus albicilla*, HAAL, circles), red kites (*Milvus milvus*, MIML, stars), and osprey (*Pandion haliaetus*, PAHA, squares) nestlings in 2019 and 2020 from Germany. White symbols indicate that the concentrations were below reporting limits (RL) in the respective species.

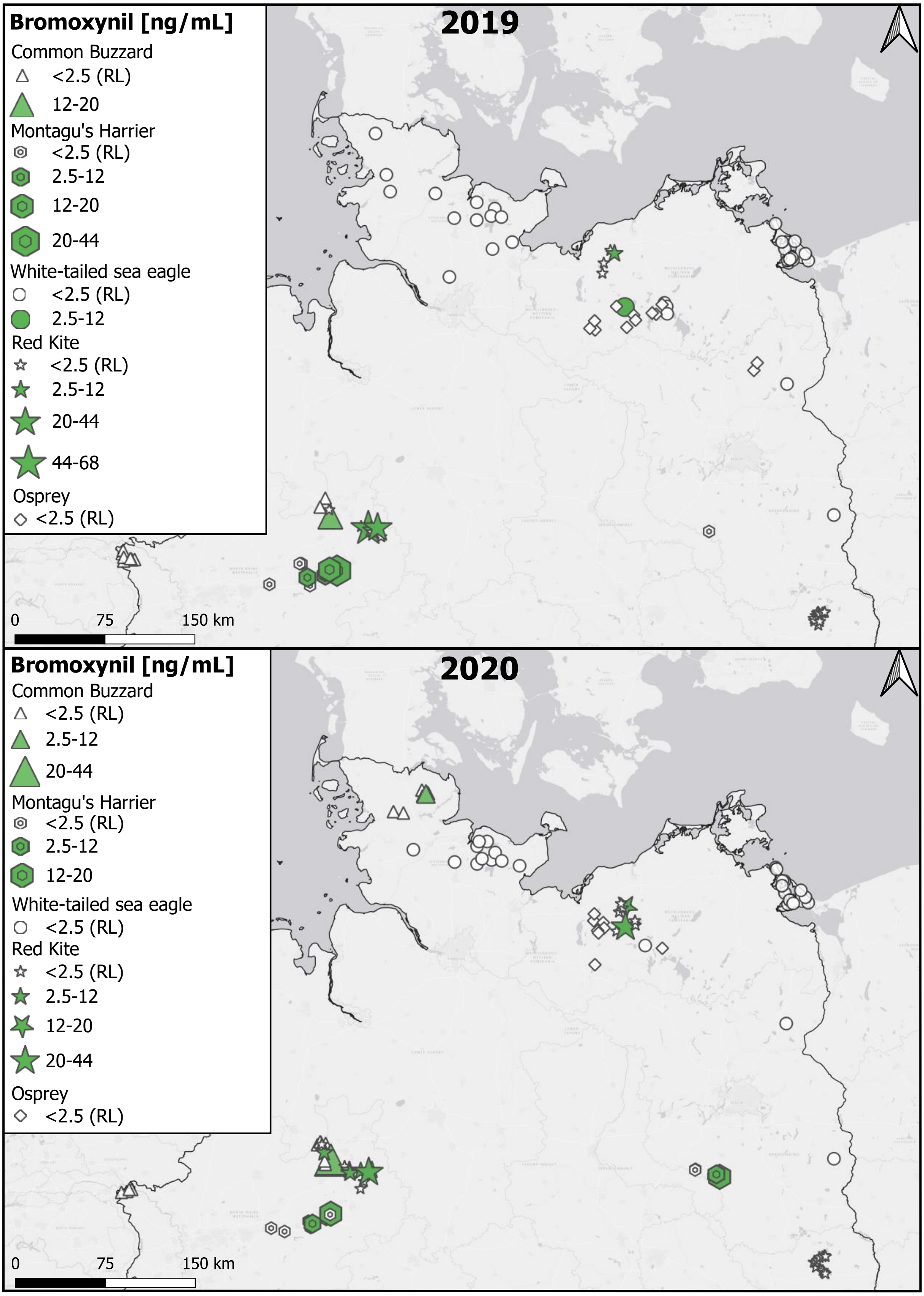

Figure SI-3: Spatial detection of bromoxynil (green) in blood of common (*Buteo buteo*, BUBT, triangles), Montagu's harriers (*Circus pygargus*, CIPY, doubled hexagons), white-tailed sea eagle (*Haliaeetus albicilla*, HAAL, circles), red kites (*Milvus milvus*, MIML, stars), and osprey (*Pandion haliaetus*, PAHA, squares) nestlings in 2019 and 2020 from Germany. White symbols indicate that the concentrations were below the reporting limit (RL) in the respective species.

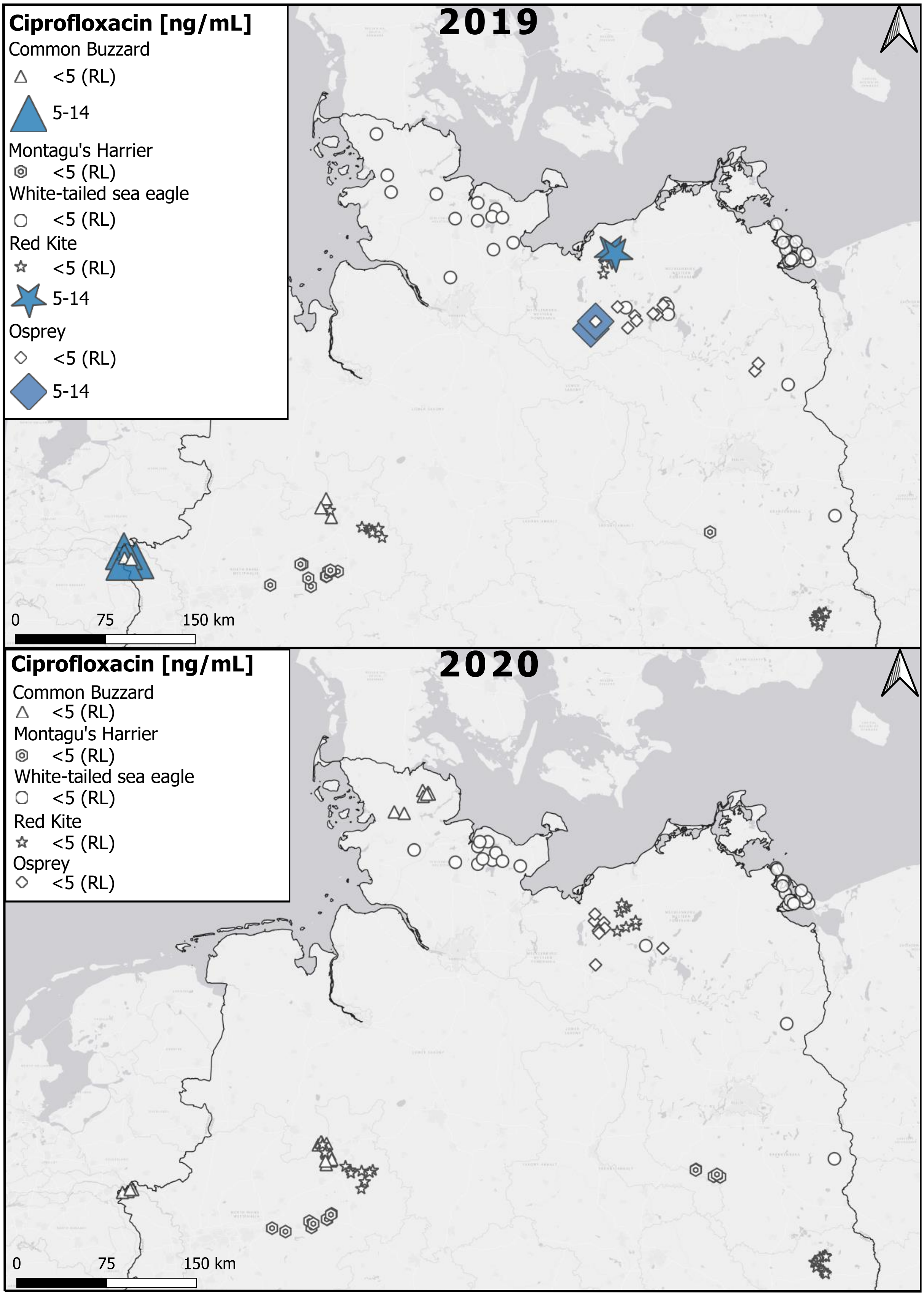

Figure SI-4: Spatial detection of and ciprofloxacin (blue) in blood common (*Buteo buteo*, BUBT, triangles), Montagu's harriers (*Circus pygargus*, CIPY, doubled hexagons), white-tailed sea eagle (*Haliaeetus albicilla*, HAAL, circles), red kites (*Milvus milvus*, MIML, stars), and osprey (*Pandion haliaetus*, PAHA, squares) nestlings in 2019 and 2020 from Germany. White symbols indicate that the concentrations were below the reporting limit (RL) in the respective species.

References

BVL. Domestic sales and export of plant protection products in 2019. 2020; Bundesamt für Verbraucherschutz und Lebensmittelsicherheit (BVL): https://www.bvl.bund.de/psmstatistiken.

EEA. Corine Land Cover (CLC) 2018, Version 20. European Environment Agency (EEA), https://land.copernicus.eu/pan-european/corine-land-cover/clc2018, 2018.

Wallmann J, Bode C, Köper L, Heberer T. In German: Abgabenmengenerfassung von Antibiotika in Deutschland 2019. Deutsches Tierärzteblatt 2020; 68 (9).
